# Protein Core Fucosylation Regulates Planarian Head Regeneration *via* Neoblast Proliferation

**DOI:** 10.3389/fcell.2021.625823

**Published:** 2021-07-16

**Authors:** Wenjun Wang, Yuan Yu, Hongbo Liu, Hanxue Zheng, Liyuan Jia, Jing Zhang, Xue Wang, Yang Yang, Fulin Chen

**Affiliations:** ^1^Lab of Tissue Engineering, College of Life Sciences, Northwest University, Xi’an, China; ^2^Provincial Key Laboratory of Biotechnology of Shaanxi, Northwest University, Xi’an, China; ^3^Key Laboratory of Resource Biology and Biotechnology in Western China, Ministry of Education, School of Medicine, Northwest University, Xi’an, China

**Keywords:** planarian, head regeneration, neoblast proliferation, FUT8, core fucose-binding glycoproteins

## Abstract

Protein glycosylation is an important posttranslational modification that plays a crucial role in cellular function. However, its biological roles in tissue regeneration remain interesting and primarily ambiguous. In this study, we profiled protein glycosylation during head regeneration in planarian *Dugesia japonica* using a lectin microarray. We found that 6 kinds of lectins showed increased signals and 16 kinds showed decreased signals. Interestingly, we found that protein core fucosylation, manifested by Lens culinaris agglutinin (LCA) staining, was significantly upregulated during planarian head regeneration. Lectin histochemistry indicated that the LCA signal was intensified within the wound and blastemal areas. Furthermore, we found that treatment with a fucosylation inhibitor, 2F-peracetyl-fucose, significantly retarded planarian head regeneration, while supplement with L-fucose could improve *DjFut8* expression and stimulate planarian head regeneration. In addition, 53 glycoproteins that bound to LCA were selectively isolated by LCA-magnetic particle conjugates and identified by LC-MS/MS, including the neoblast markers DjpiwiA, DjpiwiB, DjvlgA, and DjvlgB. Overall, our study provides direct evidence for the involvement of protein core fucosylation in planarian regeneration.

## Introduction

Planarians are flatworms that have drawn increasing interest in the scientific community due to their remarkable regenerative ability. Planarians are capable of completely regenerating any missing tissue within several days, whether amputated transversely or longitudinally, and even small fragments that have been removed from the body can regenerate into entire worms (Reddien and Sánchez Alvarado, 2004). Planarian regeneration relies on adult stem cells, called neoblasts, which are broadly distributed throughout the worm’s body (Wagner et al., 2011). After amputation, neoblasts respond to local signaling molecules and then migrate to the wound site and proliferate (Adell et al., 2010). Their descendants can form a regenerated blastema and develop into a highly organized structure to replace the amputated tissues (Wagner et al., 2011). Thus, the organism provides an excellent model for studying tissue regeneration and stem cell function.

Extensive research has focused on the genetic mechanisms underlying neoblast biology (Eisenhoffer et al., 2008; Gurley et al., 2010; Wurtzel et al., 2015). Factors found to be influencing neoblast function include fibroblast growth factor (FGF), epidermal growth factor (EGF), bone morphogenetic protein (BMP), Wnt/β-catenin, and Hedgehog signalings. It has been reported that the FGF signaling pathway controls the spatial restriction of brain tissues in the head region (Cebrià et al., 2002). EGF receptor (EGFR) signaling controls cell proliferation, differentiation, and morphogenesis (Fraguas et al., 2011). The BMP pathway regulates not only dorsoventral polarity but also mediolateral polarity (Reddien et al., 2007; Tian et al., 2021), while the Wnt/β-catenin and Hedgehog signalings control anteroposterior polarity (Gurley et al., 2008; Rink et al., 2009). Nevertheless, the transduction of these signals into the posttranslational modification (PTM) outputs is less explored and understudied.

Protein glycosylation, one of the most prominent and extensively studied PTMs, is an enzymatic process that attaches glycans to proteins and serves various functions, such as cell recognition, cell differentiation, cell proliferation, and cell migration (Wells et al., 2001). Protein core fucosylation involves the transfer of fucose from GDP-L-fucose to the innermost GlcNAc residue of an N-linked glycan on glycoproteins *via* an α1,6-fucosyltransferase (FUT8) (Wang et al., 2006). The core fucose structure can be preferentially recognized by some lectins such as LCA; thus, LCA has been often used for labeling core fucose in glycoproteins (Matsumura et al., 2007). Increasing evidence indicates that core fucosylation is closely related to several malignant tumors, including gastric, hepatic, ovarian, colorectal, and lung cancers (Hutchinson et al., 1991; Takahashi et al., 2000; Muinelo-Romay et al., 2008; Chen et al., 2013; Zhao et al., 2014). For example, the upregulation of core fucosylation has been correlated with tumor metastasis, tumor recurrence, and poor survival in patients with non-small cell lung cancer (Chen et al., 2013). Cheng et al. suggested that FUT8-mediated multidrug resistance (MDR) in human hepatocellular carcinoma (HCC) is associated with the activation of the PI3K/Akt pathway and the expression of MDR-related protein 1 (Cheng et al., 2013). Lee et al. showed that FUT8 gene was proved to be upregulated upon Wnt/β-catenin activation, and the Wnt signaling pathway is frequently deregulated in HCC (Lee et al., 2007). Zhao et al. found that the upregulated FUT8 expression in human gastric cancer cells could lead to low proliferation (Zhao et al., 2014). Previous studies have also explored the role of core fucosylation in tissue and organ regeneration. In mammals, core fucosylation is crucial for the ligand-binding affinity of TGF-βR, EGFR, and integrin α3β1 (Wang et al., 2005, 2006; Zhao et al., 2006). Lack of core fucosylation on these receptors may lead to a significant reduction in ligand-binding ability and downstream signaling. Recently, it was reported that core fucosylation was physiologically associated with the proliferation of hepatocytes and liver regeneration. Lack of core fucosylation inhibited the EGFR- and hepatocyte growth factor receptor (HGFR)-mediated signaling cascades both *in vitro* and *in vivo*, and it led to retarded liver regeneration in a mouse partial hepatectomy model (Wang et al., 2015). Collectively, these studies suggest the potential role of protein core glycosylation in tumorigenesis and tissue regeneration. However, whether core glycosylation plays a part in planarian regeneration remains ambiguous.

In this study, we first screened protein glycosylation alteration during planarian head regeneration using a lectin microarray. Then, we used 2F-peracetyl-fucose (2F-P, a fucosylation inhibitor) and L-fucose (a fucose provider), to observe the effects of protein core fucosylation during planarian head regeneration. Finally, we isolated and identified the potential core fucose-bearing protein using LCA-magnetic particle conjugates (LMPCs) and the mass spectrum database. These results demonstrated that protein core fucosylation was upregulated during planarian head regeneration and regulated the regenerative process *via* modulating neoblast proliferation.

## Materials and Methods

### Animals

A clonal strain of the planarian *Dugesia japonica* was used in all experiments. The colony was maintained in autoclaved stream water at 20∘C. The animals were shielded from light and fed with fresh chicken liver paste twice per week. The animals of 8-mm length were selected and starved for 1 week prior to all experiments. For regeneration studies, planarians were cut into two fragments (head and tail) by transverse amputation at the anterior pharynx. The whole-tail fragments including the regenerated blastema were harvested at 1, 3, 5, and 7 days post amputation (dpa), and the tail fragments amputated immediately were used as 0 dpa.

### Quantitative Real-Time PCR (qPCR) Analysis

The qPCR analysis was performed using SYBR Premix Ex Taq (TaKaRa, Beijing, China) and a CFX96^TM^ Real-Time PCR Detection System (Bio-Rad, Hercules, CA, United States) with the following parameters: 95∘C for 10 s; followed by 40 cycles at 95∘C for 5 s and 60∘C for 30 s. Total RNA was extracted with TRIzol Reagent (Invitrogen, Carlsbad, CA, United States), and reverse transcription was performed to generate cDNA using a Transcriptor First Strand cDNA Synthesis Kit (Roche, Basel, Switzerland). The β-actin housekeeping gene was selected as an endogenous reference. The relative gene expression of each gene was calculated using the 2^–ΔΔ*CT*^ method. The qPCR primers are listed in Supporting Information Supplementary Table S1.

### Immunohistochemistry

For immunohistochemistry, the samples from each time point were sacrificed with 2% HCl for approximately 5 min on ice and fixed with 4% paraformaldehyde at room temperature. For lectin histochemistry, the fixed samples were embedded by routine paraffin wax processing and serially sectioned at a width of 4 μm. The gross morphology of the regrowing planarians was observed with a dissecting microscope (Olympus, Tokyo, Japan).

### Lectin Microarray and Data Analysis

For the lectin microarray, total protein from the collected tissues was lysed using T-PER Reagent (Pierce, Thermo Fisher Scientific, Waltham, MA, United States) including 1% protease inhibitors. Thirty-seven lectins (purchased from Vector Laboratories, Burlingame, CA, United States; Sigma-Aldrich, St. Louis, MO, United States; and Calbiochem, San Diego, CA, United States) were used to conduct the lectin microarray as previously described (Qin et al., 2016). Total protein was extracted from 10 tail fragments at each time point and then labeled with Cy5 fluorescent dye (GE Healthcare, Saint Giles, United Kingdom) according to the manufacturer’s instructions. Six micrograms of Cy5-labeled protein was diluted with 0.5 ml of incubation buffer containing 2% BSA, 500 mM glycine, and 0.1% Tween-20 and incubated at 37∘C for 3 h. Finally, the microarrays were scanned with a 70% photomultiplier tube at a laser power setting of 100% with a GenePix 4000B confocal scanner (Axon Instruments, Burlingame, CA, United States). The acquired images were analyzed at 532 nm for Cy5 detection using GenePix 3.0 software (Axon Instruments, Inc., United States). The average background values were subtracted, and values that were less than the average background ± 2 standard deviations (SD) were removed from each data point to get the effective data points, and the median of the effective data points of each sample was counted. The median of the effective data points from each lectin in the block was globally normalized to the sum of the medians of all the effective data points for each lectin in one block. Each sample was observed consistently on three repeated slides, the normalized medians of each lectin from nine repeated blocks were averaged, and the SD was calculated. The normalized data from the experimental group and its control were compared to determine the relative change in protein glycosylation levels. Differences between two corresponding data sets were evaluated using Student’s *t*-test for each lectin signal using SPSS statistical software.

### Lectin Histochemistry and Immunofluorescence Detection of Phospho-Histone H3 (H3P)

For lectin histochemistry, 10 fixed samples of each time point were dehydrated in ethanol, transparentized in dimethylbenzene, embedded in paraffin, and sliced continuously into 4-μm sections. The sections were deparaffinized in gradient ethanol, antigen retrieved in 0.1 M sodium citrate buffer (pH 6.0), and blocked in 0.6% IgG-free BSA (Sigma, United States) and 0.45% fish gelatin (Sigma, United States) for 30 min at 37∘C. Next, the slides were incubated with lectins labeled with Cy5 fluorescent dye at a final concentration of 15–20 μg/ml with blocking buffer for 3 h at room temperature in the dark. After washing three times for 15 min in phosphate-buffered solution (PBS), the slides were incubated with 4′,6-diamidino-2-phenylindole (DAPI, 1 μg/ml) for 5–10 min. To assess the proliferation of neoblasts during regeneration *in vivo*, whole-mount H3P labeling was performed as previously described (Newmark and Sánchez Alvarado, 2000; Oviedo et al., 2008). After fixation, the animals were washed in methanol at 4°C for 1 h, bleached overnight in 6% H_2_O_2_, rehydrated in methanol/PBS supplemented with 0.3% Triton X-100 (PBTX) series, and blocked in blocking buffer (above) for 2–4 h while shaking. Rabbit anti-H3P (diluted 1:250 in blocking buffer, R&D Systems, Minneapolis, MN, United States) and FITC-conjugated goat anti-rabbit IgG (diluted 1:200 in blocking buffer, Millipore, Burlington, MA, United States) were used. After incubation, the samples were washed six to eight times in PBTX for 8 h. Each result was repeated at least three times in lectin histochemistry and H3P labeling. Furthermore, the images were captured with the same acquisition parameters by a laser scanning confocal microscope (FV1000, Olympus, Japan).

### Effects of Fucosylation Inhibitor on Planarian Head Regeneration

As an analog of fucose, 2F-P can penetrate cells and inhibit fucosylation. The regenerating planarians were kept in water containing 500 μM 2F-P, and samples were harvested at different time points after amputation and observed. A lectin microarray was used to verify alterations in fucosylation after treatment with 2F-P. qPCR was performed to evaluate the mRNA expression of the neoblast marker gene *DjMCM2*, a planarian *MCM2 (minichromosome maintenance 2)* homolog.

### Effects of L-Fucose Treatment on Planarian Head Regeneration

In the formation of core fucosylation, using GDP-L-fucose as a substrate, FUT8 specifically transfers L-fucose to these oligosaccharides. To determine whether L-fucose could affect head regeneration, planarians were treated with L-fucose with different concentrations. Samples were harvested 1, 3, and 5 dpa, and total RNA was extracted for qPCR assay.

### Isolation and Identification of Glycoproteins That Bound to LCA

Glycoproteins that bound to LCA were selectively isolated by LMPCs as previously described (Zhong et al., 2015). Briefly, 2 mg of total protein from regenerating planarians at different time points was diluted in 600 μl of binding buffer (0.1 M Tris–HCl, 150 mM NaCl, 1 mM CaCl_2_, 1 mM MgCl_2_, and 1 mM MnCl_2_, pH 7.4) supplemented with 1% proteinase inhibitor. The LMPCs, manufactured by the Laboratory for Functional Glycomics, were rinsed three times using binding buffer, followed by incubation with the protein samples at room temperature for 3 h with gentle shaking according to a previously published protocol (Qin et al., 2014). Then, the unbound proteins were removed by washing them three times. The glycoproteins bound to the LMPCs were eluted with 300 μl of elution buffer (8 mol/l urea and 0.1 mol/l NH_4_CO_3_) at room temperature for 1 h with gentle shaking.

### Identification of Peptides by LC-MS/MS

According to a previously described protocol, the glycoproteins were concentrated and desalted by a size-exclusion spin filter (Amicon Ultra-0.5 10 K device) with a molecular mass cutoff of 10 kDa (Yang et al., 2013). The obtained glycoproteins were denatured in 8 M urea, deoxidized with 10 mM DTT, and carboxyamidomethylated with 20 mM iodoacetamide. Subsequently, trypsin was added at a 1:100 (w/w) ratio of enzyme to protein, and the samples were incubated overnight at 37∘C. Glacial acetic acid (5 μl, pH 2.0) was added to stop the reaction, the samples were centrifuged, and the supernatant was collected and lyophilized. Then, the *N-*linked glycans of the glycopeptides were released by incubation with PNGase F overnight at 37∘C. The peptides were desalted using C18 Sep-Pak columns and lyophilized. Finally, the peptides were resolubilized in 0.1% (v/v) formic acid and analyzed using nanospray CHIP-LC-MS/MS 6530 mass spectrometers equipped with HP 1200 solvent delivery systems. Each sample was observed consistently on three replicates, and the data were automatically extracted using Agilent MassHunter Workstation software. All MS/MS spectra were searched against the International Protein Index using MASCOT 2.3.0 with the following parameters: a mass tolerance of ±20 ppm for precursor ions and a tolerance of ±0.7 Da for fragmentations. One missed cleavage was allowed. Carboxymethylated cysteines were set as a fixed modification, and oxidized methionines were set as a variable modification. MASCOT scores were above 25 for complete proteins. These criteria were a compromise to eliminate the risk of having too many false positives if the values were set too low or of missing real protein hits if the values were set too high.

### Quantitative and Statistical Analysis

Data processing, statistical analysis, and diagram were done in Excel. Cy5 detection was done using GenePix 3.0 software (Axon Instruments, Inc., United States), and differences between two corresponding data sets were evaluated using Student’s *t*-test and one-way analysis of variance (ANOVA) in SPSS statistical software with *p*^∗∗∗^ < 0.001, *p*^∗∗^ < 0.01, and *p*^∗^ < 0.05. The identified LCA-binding proteins were classified by Blast2GO (GO annotation) and then analyzed by WEGO and KEGG software.

## Results

### Alterations in Tissue Glycopatterns During Planarian Head Regeneration

**FIGURE 1:**
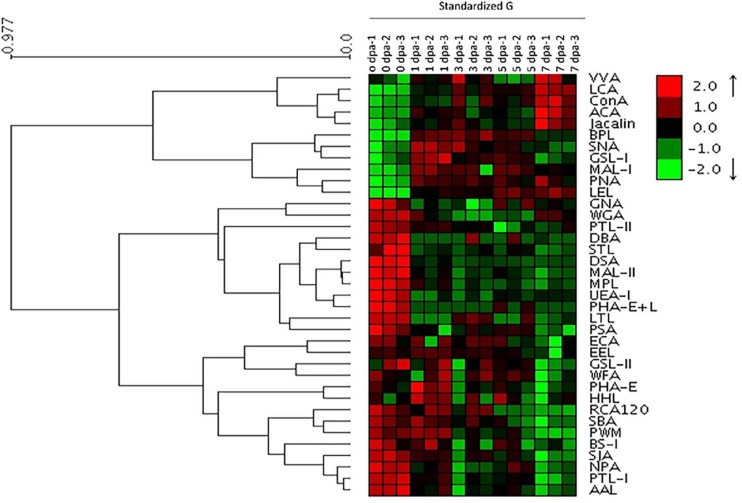
Profile of glycans targeting the head regeneration of planarians by lectin microarray. Heatmap and hierarchical cluster analysis of the 37 lectins with three biological replicates. Red and green indicate increased and decreased lectin signals, respectively.

**FIGURE 2:**
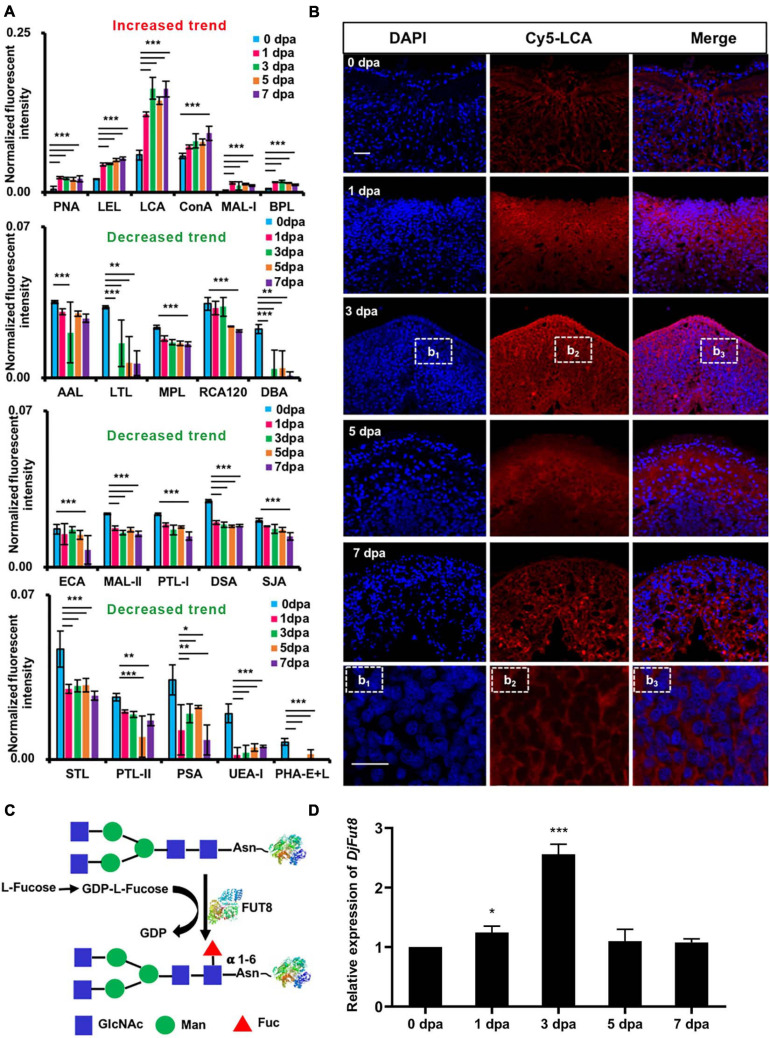
Glycans with significantly altered in the control compared with the regenerating planarians. **(A)** Normalized fluorescent intensity of lectins with altered signals in planarian head regeneration from lectin microarray. Six increased (ratio (1–7 dpa/0 dpa) > 1.5) and 15 decreased (ratio (1–7 dpa/0 dpa) < 0.67) lectin signals compared with control (*p < 0.05, **p < 0.01, ***p < 0.001). **(B)** Double-labeling of LCA-binding glycan core fucoses increased during the regeneration process (blue labeling of nuclei by DAPI; red labeling of the perinuclear cytoplasm and the cell membrane by LCA) compared with the control. The letters b_1_, b_2_, and b_3_ indicate magnified images of dotted frames in sections at 3 dpa. Scale bars = 10 μm **(C)** Synthesis of α 1,6-fucose (core fucose). **(D)** qPCR showed that *DjFut8* expression was upregulated at 1 dpa and 3 dpa (*p < 0.05, ***p < 0.001).

Lectin microarray chips containing 37 lectins were used to detect alterations in glycosylation during planarian head regeneration. Supplementary Figure 1A portrays the sketch of planarian head regeneration. The layout of the lectin microarray and glycopatterns of glycoproteins during planarian head regeneration are shown in Supplementary Figures 1B,C. The data generated from each regeneration time point were analyzed using Expander 6.0. By hierarchical cluster analysis, the lectins were clustered into two major groups. Heatmap results further visually demonstrated that lectin signals in the upper group (from VVA to LEL) exhibited an increasing trend, while lectin signals in the lower group (from PTL-II to AAL) exhibited a decreased trend during planarian regeneration (Figure 1). The different trends between the two clusters were verified by quantification analysis of the normalized fluorescence intensities, and the results suggested that the majority of the lectin signals were significantly altered in their corresponding clusters. For example, signals of LCA, ConA, BPL, MAL-1, PNA, and LEL in the upper group exhibited a significant increase [ratio (1–7 dpa/0 dpa) > 1.5], while signals of PTL-II, DBA, STL, DSA, MAL-II, MPL, UEA-I, PHA-E + L, LTL, PSA, ECA, RCA120, SIA, PTL-I, and AAL in the lower group exhibited a significant decrease [ratio (1–7 dpa/0 dpa) < 0.67] during the regenerative process (Supplementary Table S2). Figure 2A shows the significantly increased and decreased lectin signals.

Consistent with the results of the lectin microarray, lectin histochemistry indicated that, compared with control, the perinuclear cytoplasm and the membrane of the regenerating area in the specimens showed increased LCA staining of α1,6-fucose (core fucose) (Figure 2B). In addition, the mRNA expression levels of *Dugesia japonica* α1,6-fucosyltransferase (*DjFut8*, homology of α1,6-fucosyltransferase), which catalyzes core fucosylation synthesis (Figure 2C), were upregulated at 1 dpa (^∗^*p* < 0.05) and 3 dpa (^∗∗∗^*p* < 0.001) (Figure 2D).

### Fucosylation Inhibitor Retards Planarian Head Regeneration

**FIGURE 3:**
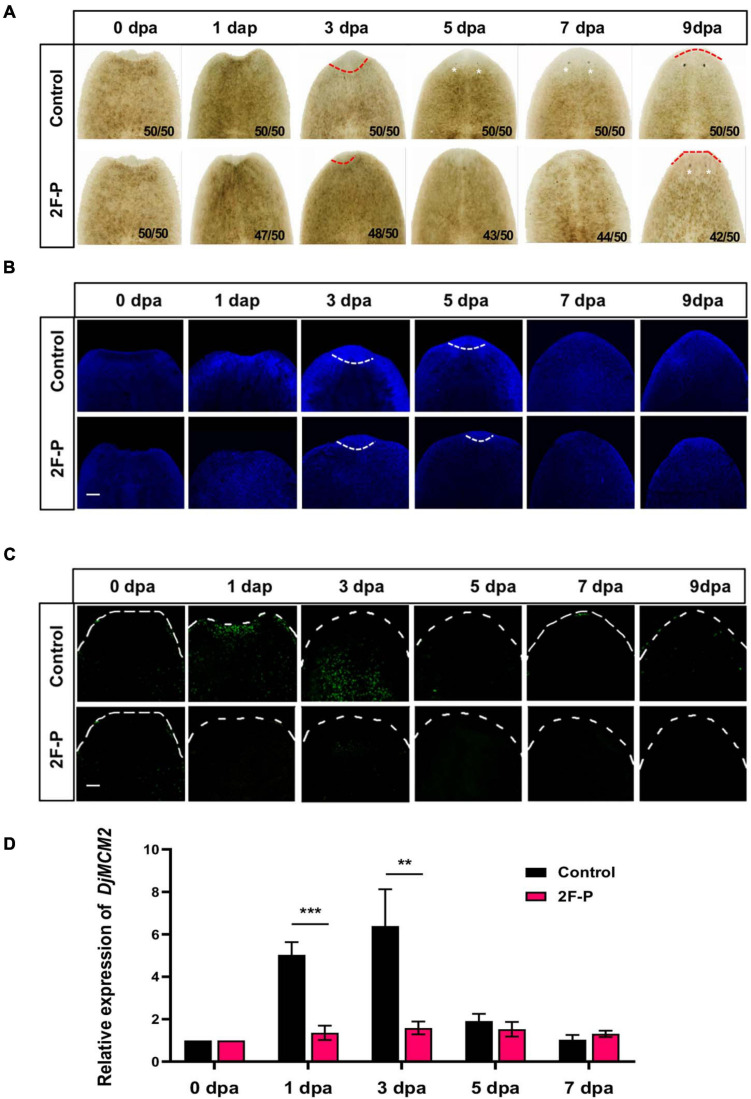
Effect of fucosylation inhibitor 2F-P on planarian head regeneration. **(A)** Treatment with fucosylation inhibitor 2F-P retarded head regeneration; eye spot formation was noted at 5 dpa in control (white arrow) and at 9 dpa after treatment with 2F-P. **(B)** Whole-mount DAPI labeling. Increased cell density was observed at 1 and 3 dpa in control, and slightly decreased cell density was detected after treatment with 2F-P at the wound trunk fragment (white dotted line). Scale bars = 100 μm. **(C)** The accumulation of H3P-positive cells was observed at 1 and 3 dpa in control (white arrow), and slightly decreased cell density was detected at 7 and 9 dpa after treatment with 2F-P at the wound trunk fragment. Scale bars = 100 μm. **(D)** qPCR demonstrated the inhibition of *DjMCM2* expression at 1 dpa (***p < 0.001) and 3 dpa (**p < 0.01) between control and treatment with 2F-P specimens during head regeneration.

Figure 3A displays the *Dugesia japonica* head regeneration process after anterior pharynx amputation. The formation of eye spots could be observed as early as 5 dpa, and the animals completely regenerated their heads at 7 dpa. DAPI (4′,6-diamidino-2-phenylindole) labeling results showed that cell density increased significantly in the wound area at 1 dpa and 3 dpa (Figure 3B). The accumulated cells in the regenerating area were positive for H3P staining in the 1- and 3-dpa specimens (Figure 3C). Because neoblasts are the only proliferative cells in the planarian body, phospho-histone H3 (H3P) labeling indicates the proliferative neoblasts (Wenemoser and Reddien, 2010). We then used the fucosylation inhibitor 2F-P to explore the functions of fucosylation in planarian head regeneration. The lectin microarray indicated that 2F-P could significantly inhibit LCA signals (^∗∗∗^*p* < 0.001), as shown in Supplementary Figures 1D,E. Consequently, planarian head regeneration was retarded, the eye spots formed at 9 dpa (Figure 3A), and cell proliferation was also inhibited (Figure 3C). In addition, we tested the expression of a neoblast proliferation marker, *DjMCM2* (Salvetti et al., 2000). As shown in Figure 3D, *DjMCM2* expression was increased at 1 dpa and peaked at 3 dpa in control regenerative animals, while its expression was significantly inhibited at 1 dpa (^∗∗∗^*p* < 0.001) and 3 dpa (^∗∗^*p* < 0.01) by treatment with 2F-P. To confirm the effective time window of 2F-P inhibition, planarians were allowed to regenerate in water supplemented with 2F-P during the whole regenerative period (Sustained inhibition), from 0 to 3 dpa (Early inhibition), or from 3 to 9 dpa (Late inhibition, Supplementary Figure 2A). Supplementary Figure 2B displays that eye spots became visible at 5 dpa in control animals. Meanwhile, the animals in the Sustained and Early inhibition groups did not regenerate eye spots, while those in the Late inhibition group formed eye spots at 5 dpa. Nevertheless, all 2F-P-treated animals finally regenerated their eyes at 9 dpa. These data suggested that 2F-P mainly exerted its inhibitory effect at the early stage during planarian regeneration, the time window when *DjFut8* is highly expressed (Figure 2D).

### L-Fucose Treatment Stimulates Planarian Head Regeneration

**FIGURE 4:**
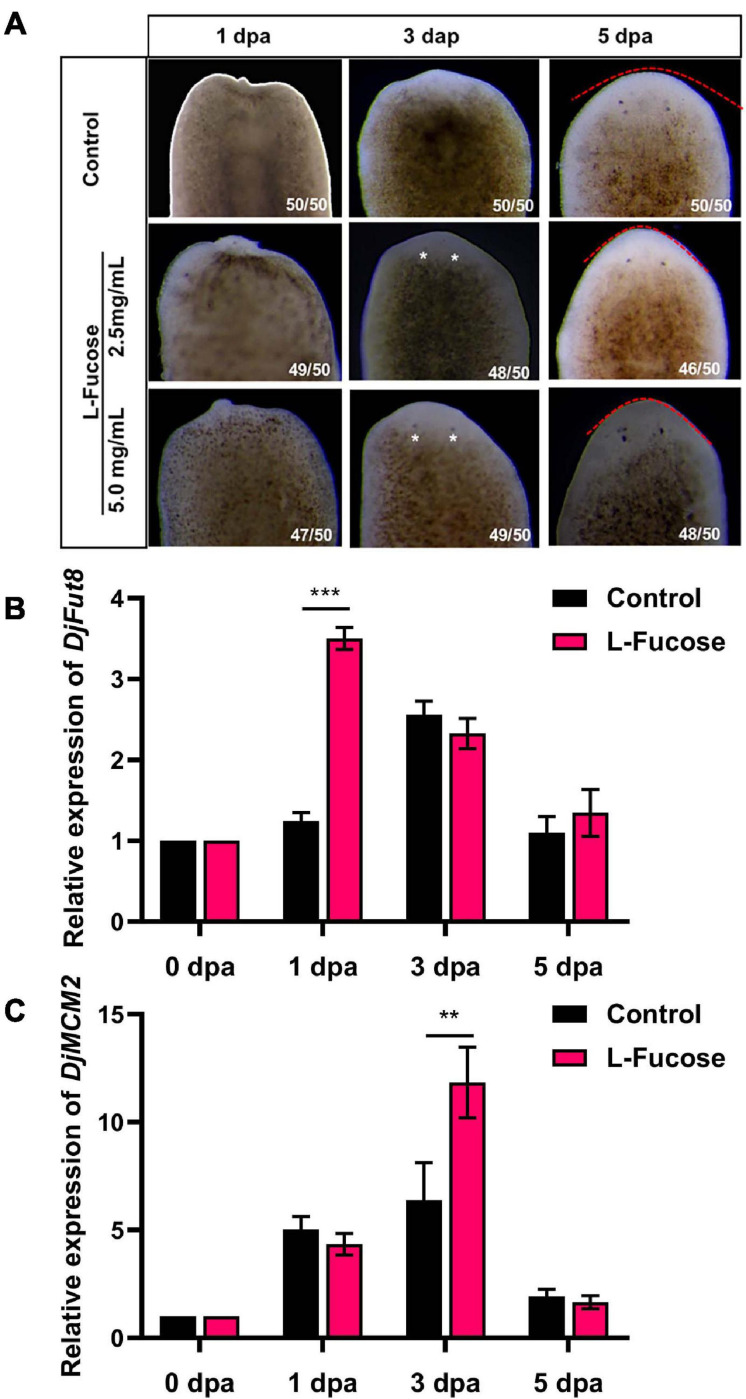
Effect of L-Fucose on planarian head regeneration. **(A)** Visible eyespots were observed at 3 dpa (white arrow) after treatment with L-fucose and at 5 dpa in control. The eye spots were more noticeable in the 5-mg/ml group than in the 2.5-mg/ml group. **(B)** and **(C)** qPCR showed the improved *DjFut8* expression at 1 dpa (***p < 0.001) and *DjMCM2* expression at 3 dpa (**p < 0.01) between the control and supplement with 5 mg/ml L-fucose specimens during head regeneration.

To assess the effect of L-fucose on planarian head regeneration, the amputated animals were treated with L-fucose. Figure 4A shows that L-fucose could accelerate planarian head regeneration at the concentration of 2.5 or 5 mg/ml. Planarians regenerated visible eye spots at 3 dpa after supplement with L-fucose while eye spots could be observed at 5 dpa in control animals. The qPCR results showed that the expression of *DjFut8* mRNA was upregulated in the L-fucose-treated animals at 1 dpa compared with control animals (^∗∗∗^*p* < 0.001, Figure 4B). Additionally, the expression level of *DjMCM2* mRNA was remarkably elevated at 3 dpa (^∗∗^*p* < 0.01, Figure 4C), indicating that L-fucose treatment might stimulate planarian head regeneration *via* neoblast proliferation.

### Identification of LCA-Binding Glycoproteins During Planarian Head Regeneration

**FIGURE 5:**
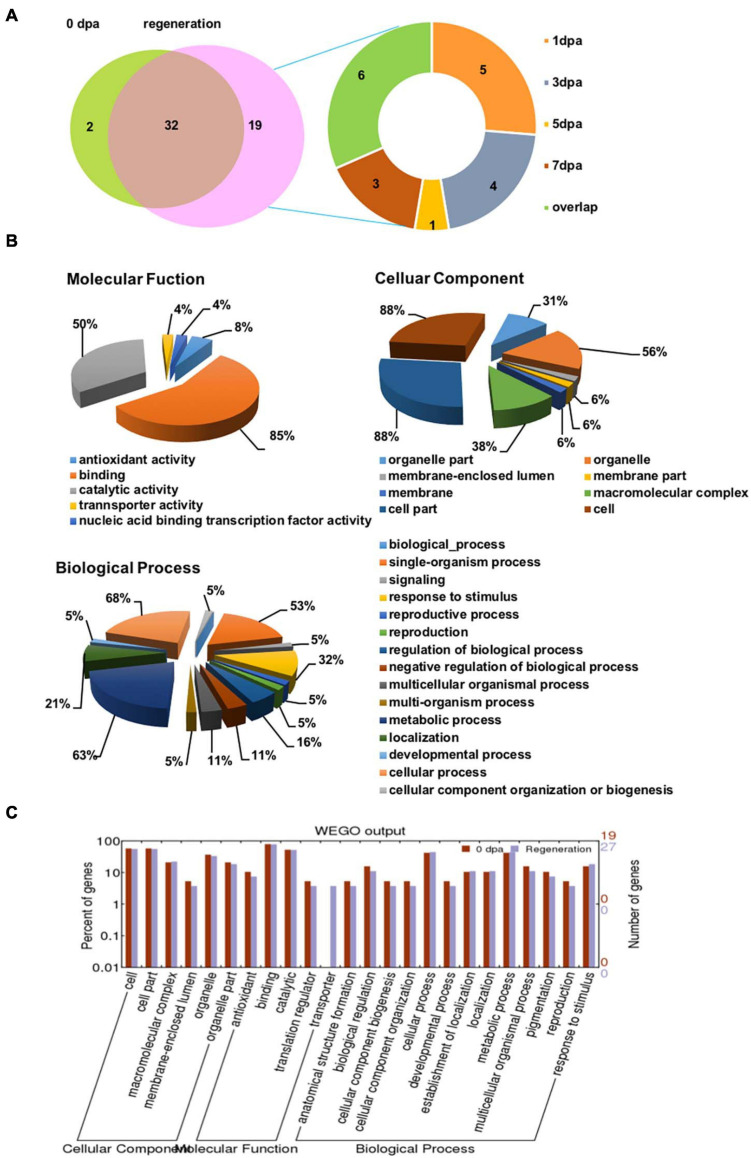
LC-MS/MS identification and GO annotations of the isolated LCA-binding glycoproteins during planarian head regeneration. **(A)** The Venn diagrams present the numbers of LCA-binding glycoproteins identified by LC-MS/MS. Left: the identified LCA-binding glycoprotein numbers in the control and planarian regenerating tissue. Right: profiles of LCA-binding glycoproteins in the control and planarian regenerating tissue of tissue at different dpa. **(B)** Total LCA-binding glycoproteins identified during planarian head regeneration were analyzed for functional enrichment according to three grouping classifications: cellular component, biological process, and molecular function with the software Blast2GO. **(C)** Gene ontology (GO) classification and comparison of enrichment of functional groups between the control and regenerating specimens using WEGO software.

The glycoproteins that bound to LCA were identified by LC-MS/MS. A total of 34/53 and 51/53 glycoproteins were identified from the regenerating planarian at 0 dpa and the rest regeneration period, respectively (Figure 5A, left). Furthermore, only two glycoproteins were exclusively identified in the animals at 0 dpa, while 19 glycoproteins were exclusively detected in the regenerating animals, among which 5/53 (9.4%), 4/53 (7.5%), 1/53 (1.9%), and 3/53 (5.7%) glycoproteins were exclusively identified in the regenerating animals at 1, 3 dpa, 5, and 7 dpa, respectively. The names of the detected LCA-binding glycoproteins and their exact expression profiles are listed in Supporting Information Supplementary Table S3.

### GO Classification of the Identified LCA-Binding Glycoproteins Using Blast2GO

To explore the major biological functions of LCA-binding glycoproteins during head regeneration, Blast2GO was used to analyze the glycoproteins according to grouping classifications, which included molecular functions, cellular components, and biological processes. Among 53 LCA-binding glycoproteins, 28 had available GO annotations, and the annotated glycoproteins were mostly involved as follows: binding (85%) and catalytic activity (50%) in Molecular Function GO term; cell (88%), cell part (88%), and organelle (56%) in Cellular Component GO term; and cellular process (68%), metabolic process (63%), and single-organism process (53%) in Biological Processes GO term (Figure 5B).

To determine whether there were any differences in the three aspects of the GO annotations between control and regrowing planarians, a GO enrichment analysis was performed using WEGO software (Figure 5C). The results revealed that the functional interpretations of the control and the regenerating planarians were similar, except for those in the transporter group. Details of the GO annotation of the differentially expressed proteins are provided in Supporting Information Supplementary Table S4.

### KEGG Pathway Analysis and Functional Protein Association Networks

To identify the biological pathways involved in planarian head regeneration, we mapped the annotated sequences to the referenced canonical pathways in the Kyoto Encyclopedia of Genes and Genomes (KEGG). The identified KEGG pathways included the dorsoventral axis formation pathway (Grk/Egfr), the Wnt pathway, the cell adhesion molecule pathway, and others (Supplementary Figure 3). The identified proteins involved in the Wnt pathway (e.g., Wnt protein) were specifically detected in 0 dpa, while proteins related to the cell adhesion molecule pathway (e.g., NCAM and NRCAM) were detected specifically in the regenerating groups (Supplementary Table S3). In addition, piwi family members (DjpiwiA and DjpiwiB) involved in the dorsoventral axis formation pathway (Grk/Egfr) were detected in animals at 0 dpa and the rest of the regeneration period (Supplementary Table S3). The KEGG pathways are listed in Supporting Information Supplementary Table S5.

## Discussion

It is expected that many cellular functions are glycosylation dependent. Structural analyses by mass spectrometry have demonstrated that methylated high-mannose-type glycans are the most abundant structure in *Dugesia japonica*, and many of the major glycans from planarians have novel structures (Natsuka et al., 2011; Paschinger et al., 2011). However, the alteration and regulation of protein glycosylation during planarian head regeneration remain unclear. Lectins can specifically bind to glycans, and lectin microarray represents a rapid and valid approach to study glycoproteins (Hirabayashi et al., 2013). In this study, we profiled glycosylation patterns during planarian regeneration using a lectin microarray chip. The results suggest that glycosylation is markedly altered during the regenerative process (Figures 1, 2A). The global alteration in glycosylation may regulate the response of cells to their microenvironment, including the extracellular matrix and growth factors, after amputation.

Fucosylation is the attachment of a fucose residue to *N*-glycans, *O*-glycans, and glycolipids, and it is catalyzed by a family of enzymes called fucosyltransferases (de Vries et al., 2001). Among these, FUT8 is the only enzyme that catalyzes the transfer of fucose from guanosine diphosphate (GDP)-fucose to the innermost GlcNAc residue *via* α1,6-linkage to form a core-fucosylated glycoprotein (Yang and Wang, 2016). The upregulation of FUT8 activity and global fucosylation has been observed in malignant tumors, including hepatic, lung, thyroid, and colorectal cancer, and is closely related to the severity of these cancers by affecting tumor size, metastases, invasion, and prognosis (Yue et al., 2016). We found that the core fucosylation manifested by the LCA signal was significantly upregulated, while some terminal fucosylations, indicated by UEA-1 and LTL signals, were downregulated during planarian head regeneration (Figures 1, 2A), which is consistent with previous findings from research on liver cancer. The results of LCA lectin histochemistry showed core fucosylation occurring within the wound and blastemal areas (Figure 2B). However, it should be noted that although LCA prefers to bind to core fucose, it also binds to high mannose (Tateno et al., 2009).

As an analog of L-fucose, 2F-P penetrates cells and is converted into GDP-2F-fucose *via* the salvage pathway. GDP-2F-fucose serves as a competitive inhibitor against fucosyltransferases by stimulating the accumulation of natural GDP-fucose in the cytoplasm, which gives rise to feedback inhibition of the *de novo* synthesis of GDP-fucose (Rillahan et al., 2012). In this study, it was found that 2F-P treatment retarded planarian head regeneration. Interestingly, an early and narrow period (0–3 dpa) of 2F-P treatment also contributed to a similar regenerative phenotype, implying that 2F-P exerts its effect early in planarian regeneration. Coincidentally, the levels of core fucosylation and *DjFut8* expression were upregulated at the early stage (Figure 2), when a biphasic mitotic response of neoblasts occurs and proves to be essential for planarian regeneration (Wenemoser and Reddien, 2010). Given that 2F-P could abate the early mitotic response (Figures 3C,D) as well as the upregulation of core fucosylation (Supplementary Figures 1D,E), we thereby speculate that 2F-P impairs neoblast proliferation *via* inhibiting core fucosylation, which eventually contributes to the delayed regenerative phenotype. Our hypothesis was partially confirmed by another experiment that supplement with L-fucose could stimulate planarian head regeneration as well as upregulation of *DjFut8* and *DjMCM2* expression (Figure 4). However, 2F-P serves as a pan-fucosyltransferase inhibitor; the specific role and mechanisms of FUT8 in planarian regeneration should be evaluated in the future by more experiments such as RNAi.

To explore the function of core fucosylated proteins and understand the downstream mechanism in response to amputation, LMPCs were applied to isolate and enrich potential core fucosylated proteins for mass spectrum analysis. A total of 53 LCA-binding proteins were identified during planarian head regeneration, including neoblast markers such as DjpiwiA and DjpiwiB (Tharp and Bortvin, 2016). Moreover, DjpiwiA and DjpiwiB (Aub protein) are related to the Grk/Egfr pathway (Supplementary Figure 3A), suggesting that DjpiwiA and DjpiwiB might regulate neoblast proliferation *via* the Grk/Egfr pathway. Other than the piwi family members, many LCA-binding glycoproteins identified in this study are closely related to neoblast biology. For example, the vas-related proteins DjvlgA and DjvlgB identified at 1 and 3 dpa are involved in the totipotency of the neoblast (Shibata et al., 1999); the membrane raft-associated proteins DjFlotillin-1 and DjFlotillin-2 regulate planarian regeneration and homeostasis *via* controlling neoblast proliferation (Dong et al., 2019); mortalin, a member of the HSP70 family, maintains neoblasts in a mitotically active condition to constantly produce lineage-specific progenitors that will support tissue regeneration and physiological turnover (Conte et al., 2009). Interestingly, some LCA-binding glycoproteins prove to be key regulators of planarian neurogenesis, including tyramine beta-hydroxylase (Nishimura et al., 2008), clathrin heavy chain (Inoue et al., 2007), tryptophan hydroxylase (Lambrus et al., 2015), puromycin-sensitive aminopeptidase (Wu et al., 2017), and the cell adhesion molecule protein DjCAM (Fusaoka et al., 2006). Fusaoka et al. have well described the structure and function of neural cell adhesion molecules in planarian, and they find that *DjCAM* knockdown leads to impairment of axon fasciculation (Fusaoka et al., 2006). Consistently, in our study, KEGG analysis showed that DjCAM (NCAM/NRCAM) are involved in the neural system (Supplementary Figure 3C), implying that core-fucosylated DjCAM might be responsible for the regeneration of the nervous system. However, LCA shows a high affinity to bi-antennary N-glycans with core fucose; thus, the LCA-binding glycoproteins isolated by LMPCs in this study probably lack extended modifications of that core fucosylation, such as galactosylated and methylated core fucosylation, which have been observed in the planarian *Dugesia japonica* (Paschinger et al., 2011). It is well established that the majority of glycoproteins are membrane and secretory proteins; however, the LCA-binding glycoproteins identified in this study contain cytoplasmic and nuclear proteins, raising the possibility that some cytosolic proteins showing high affinity to core fucose-bearing glycoproteins were non-specifically pulled down by LMPCs and identified as false-positive results in this study. Therefore, further investigation should be focused on these cytosolic glycoprotein candidates to check if they are truly glycosylated.

Planarian head regeneration is a complicated process involving proper regulation of neoblast behaviors. In this study, we profiled protein glycosylation patterns during planarian head regeneration using a lectin microarray. Among the significantly altered lectins, LCA that preferentially recognized core fucose showed increased signals. Furthermore, the fucosylation inhibitor 2F-P largely retarded planarian head regeneration and neoblast proliferation, while supplement with L-fucose could improve *DjFut8* expression and stimulate the regenerative process. Finally, we isolated and identified potential core fucose-bearing proteins using LCA-magnetic particle conjugates. Collectively, these results highlight the importance of protein core fucosylation in the regulation of planarian head regeneration and neoblast proliferation.

## Data Availability Statement

The original contributions presented in the study are included in the article/Supplementary Material, further inquiries can be directed to the corresponding authors.

## Author Contributions

FC designed the research project. HL, HZ, XW, and JZ obtained and raised the animals. WW, YYu, HL, HZ, and LJ performed or supervised the laboratory work. WW, JZ, and XW did the data processing and quality control. WW, HL, and HZ analyzed the data. WW, YYa, and FC wrote the manuscript. All the authors discussed, critically revised, and approved the final version of the manuscript. All authors contributed to the article and approved the submitted version.

## Conflict of Interest

The authors declare that the research was conducted in the absence of any commercial or financial relationships that could be construed as a potential conflict of interest.

## Supplementary Material

Supplementary Figure 1 Lectin microarray on planarian head regeneration. **(A)** Sketch of planarian head regeneration. **(B)** The layout of the lectin microarray chip. Cy5-labeled BSA was spotted as a marker, and unlabeled BSA was spotted as a negative control. **(C)** Scanned fluorescent images from control and specimens at different regeneration time points. Images were scanned at 70% using a photomultiplier tube and laser power settings at 100% with a Genepix 4000B confocal scanner. Red and white frames indicate increased and decreased lectin signals, respectively, during the head regeneration process. **(D,E)** Scanned lectin microarray images and normalized LCA fluorescence intensity between the control and 2F-P treated animals at the indicated regeneration time points during head regeneration (^∗∗∗^*p* < 0.001).

Supplementary Figure 2 The effective window of 2F-P inhibition. **(A)** Experiment scheme. Planarians were allowed to regenerate in water supplemented with 2F-P during the whole regenerative period or a narrower time window. **(B)** Effect of the different treatment schemes on planarian head regeneration. It should be noted that eye spots (indicated by white arrows) became visible at 5 dpa in control animals. Bar = 1 mm.

Supplementary Figure 3 KEGG pathway analysis and functional protein association networks. The identified LCA-binding glycoproteins mapped to the KEGG pathways of the dorso-ventral axis formation **(A)**, Wnt signaling pathway **(B)**, and cell adhesion molecules **(C)**. The identified LCA-binding glycoproteins involved in these networks are labeled with green and red frames.

## Abbreviations

DjMCM2: Dugesia japonica minichromosome maintenance 2
FUT8: α 1,6-fucosyltransferase
LMPCs: LCA-Magnetic Particle Conjugates
2F-P: 2-Fluoro-peracetyl-fucose.
