# Supplementary figures and images for: Protein Core Fucosylation Regulates Planarian Head Regeneration via Neoblast Proliferation
Source: Front Cell Dev Biol. 2021 Jul 16;9:625823. doi: 10.3389/fcell.2021.625823 (PMC8322617; doi:10.3389/fcell.2021.625823)

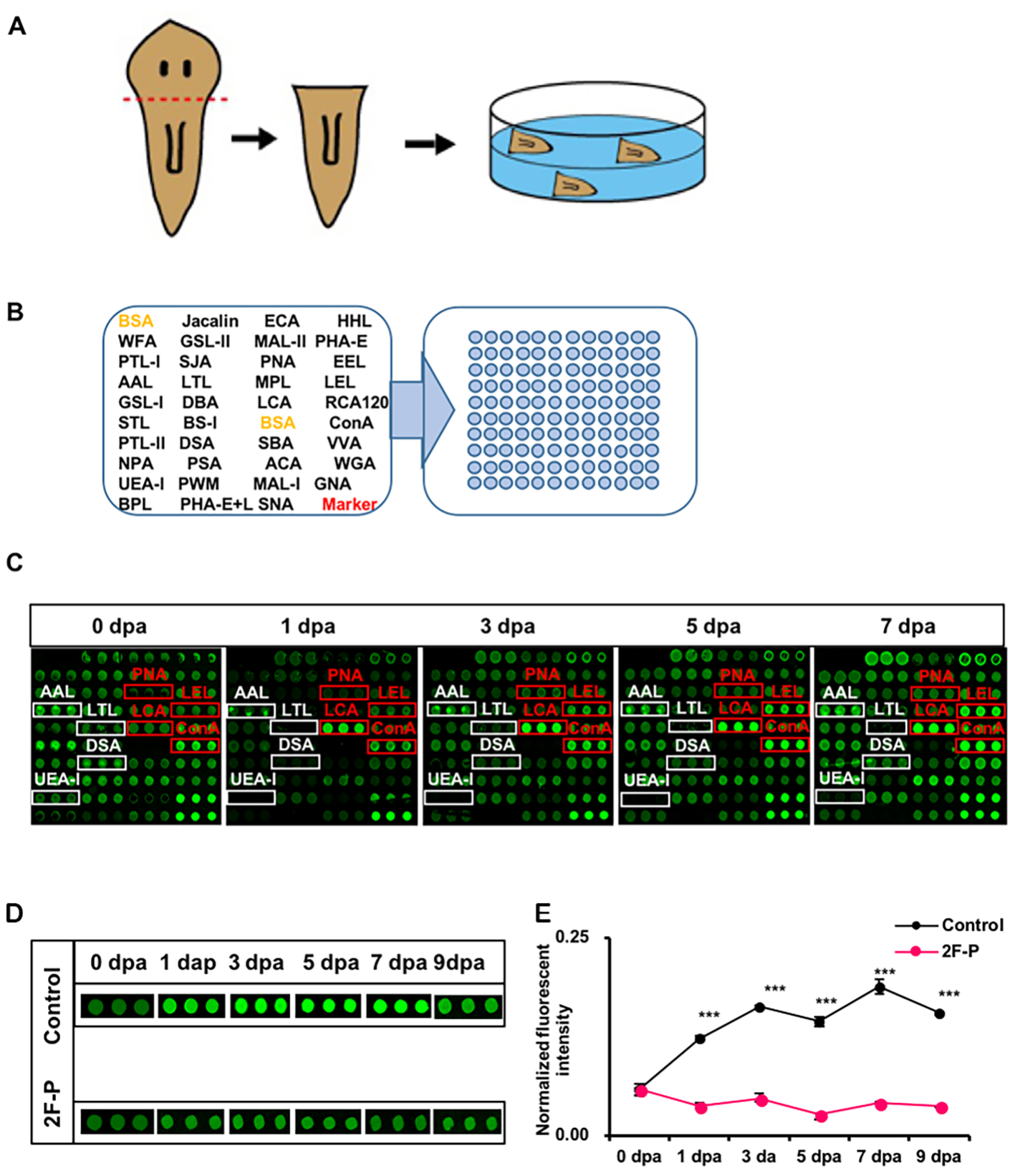

Supplement: Supplementary Figure 1 — Lectin microarray on planarian head regeneration. (A) Sketch of planarian head regeneration. (B) The layout of the lectin microarray chip. Cy5-labeled BSA was spotted as a marker, and unlabeled BSA was spotted as a negative control. (C) Scanned fluorescent images from control and specimens at different regeneration time points. Images were scanned at 70% using a photomultiplier tube and laser power settings at 100% with a Genepix 4000B confocal scanner. Red and white frames indicate increased and decreased lectin signals, respectively, during the head regeneration process. (D,E) Scanned lectin microarray images and normalized LCA fluorescence intensity between the control and 2F-P treated animals at the indicated regeneration time points during head regeneration (∗∗∗p < 0.001). [file Image_1.TIF]

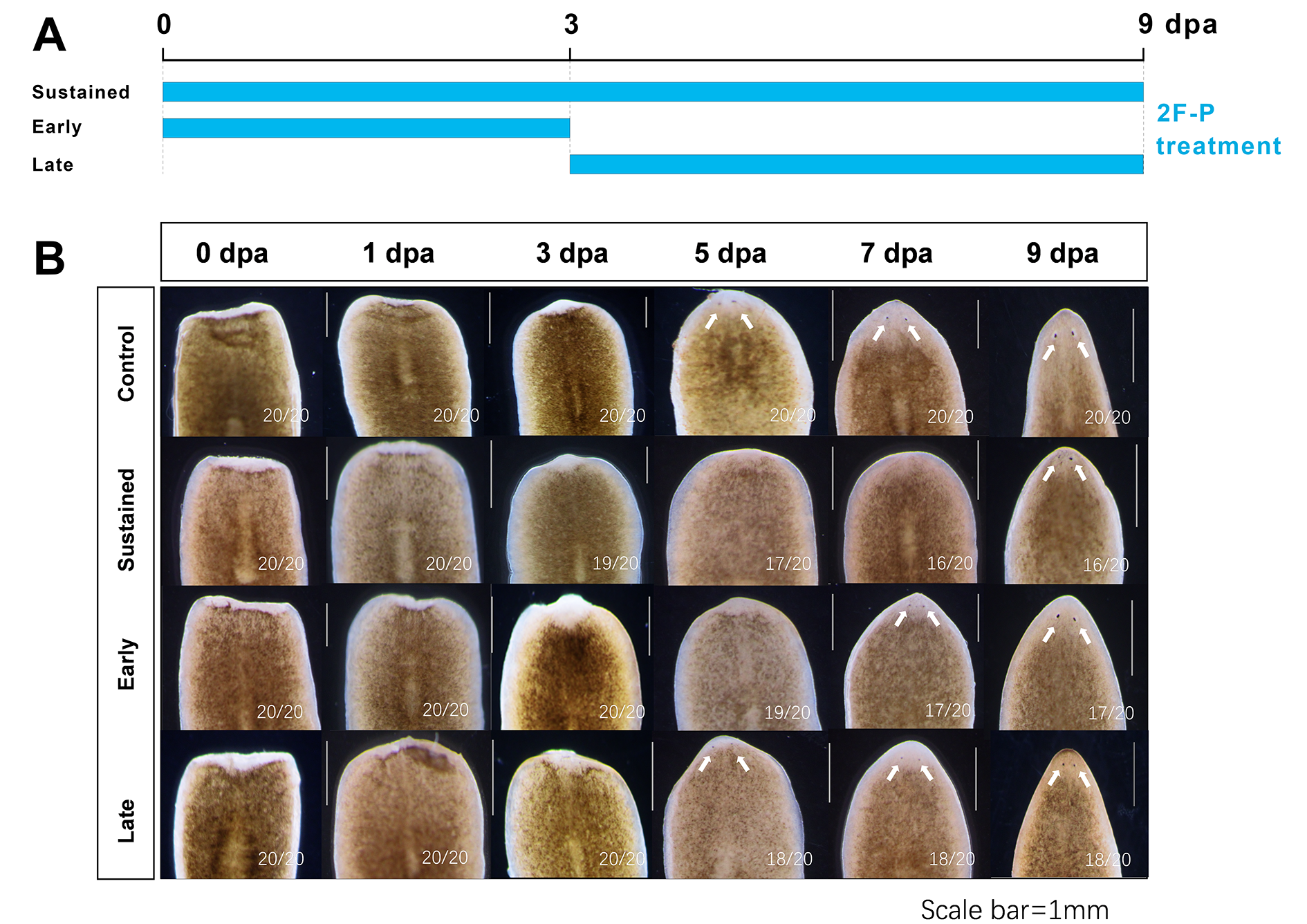

Supplement: Supplementary Figure 2 — The effective window of 2F-P inhibition. (A) Experiment scheme. Planarians were allowed to regenerate in water supplemented with 2F-P during the whole regenerative period or a narrower time window. (B) Effect of the different treatment schemes on planarian head regeneration. It should be noted that eye spots (indicated by white arrows) became visible at 5 dpa in control animals. Bar = 1 mm. [file Image_2.TIF]

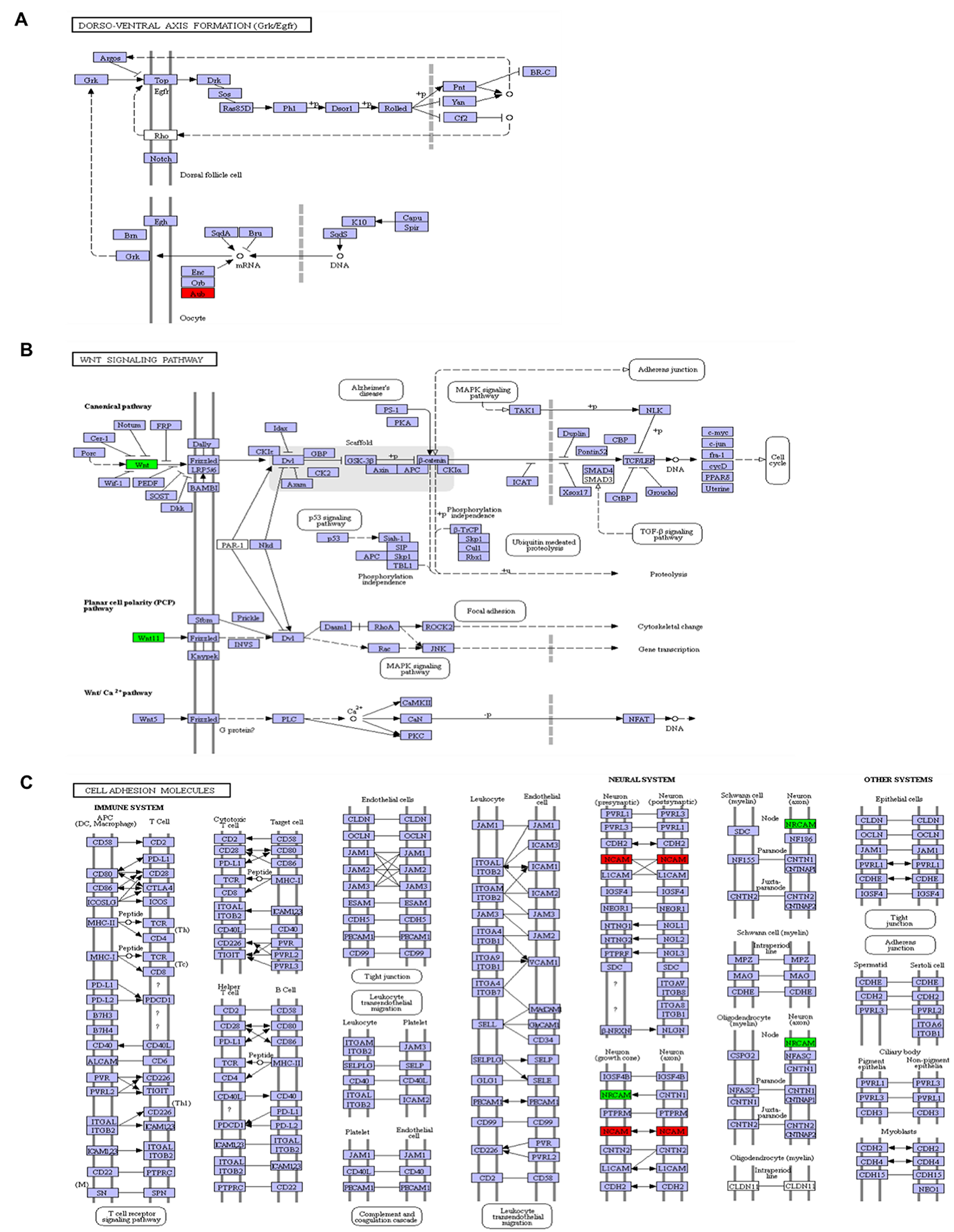

Supplement: Supplementary Figure 3 — KEGG pathway analysis and functional protein association networks. The identified LCA-binding glycoproteins mapped to the KEGG pathways of the dorso-ventral axis formation (A), Wnt signaling pathway (B), and cell adhesion molecules (C). The identified LCA-binding glycoproteins involved in these networks are labeled with green and red frames. [file Image_3.TIF]
