# Supplementary material for: Protein Core Fucosylation Regulates Planarian Head Regeneration via Neoblast Proliferation
Source: Front Cell Dev Biol. 2021 Jul 16;9:625823. doi: 10.3389/fcell.2021.625823 (PMC8322617; doi:10.3389/fcell.2021.625823)
Supplement: Supplementary file 4 [file Data_Sheet_1.docx]

# Supplementary Tables

**Table S1** Primers used in qPCR analysis.

| **Target Gene** | **Primer Sequence (5' to 3')** |
| --- | --- |
| *Beta-actin* | Forward: CGGGTCGAACAACTGGTATT |
|  | Reverse: GTGGAAGAGCGTCTTCATAG |
| *DjMCM2* | Forward: GGAGAAGAAGGAAGTGGTGAAA |
|  | Reverse: CTCGCTGTCTCGCGTAAAT |
| *DjFut8* | Forward: TCAGAAGCGAACATATCACAGT |
|  | Reverse: CAACCAAATCCGCATCCT |

Note:

*^1^ DjMCM2:* planarian *Dugesia japonica* minichromosome maintenance 2.

*^2^ DjFut8:* planarian *Dugesia japonica* α1,6-fucosyltransferase.

**Table S2** Differential glycopatterns of glycoproteins during head regeneration of planarians by lectin microarray analysis.

| **Lectin** | | **Specificity** | **Normalized fluorescence intensity (NFI) NFI±SD** | | | | | | **ratio (p-value)** | | | |
| --- | --- | --- | --- | --- | --- | --- | --- | --- | --- | --- | --- | --- |
|  |  |  | **0 dpa** | **1 dpa** | | **3 dpa** | **5 dpa** | **7 dpa** | **1 dpa/0 dpa** | **3 dpa/0 dpa** | **5 dpa/0 dpa** | **7 dpa/0 dpa** |
| **Increased lectin signals** | | | | | | | | | | | | |
| PNA | | Galβ1-3GalNAcα-Ser/Thr(T) | 0.005±0.004 | 0.023±0.001 | 0.022±0.001 | | 0.021±0.003 | 0.021±0.005 | 4.49*** | 4.24*** | 3.99*** | 4.13*** |
| LEL | | (GlcNAc)n, high-mannose-type N-glycans | 0.021±0.001 | 0.044±0.002 | 0.045±0.001 | | 0.051±0.003 | 0.053±0.003 | 2.08*** | 2.12*** | 2.41*** | 2.51*** |
| LCA | | Fucα-1,6GlcNAc | 0.058±0.007 | 0.123±0.004 | 0.148±0.017 | | 0.144±0.006 | 0.183±0.013 | 2.1*** | 2.53*** | 2.46*** | 3.13*** |
| ConA | | High-mannose,terminal GlcNAc,Manα1-6(Manα1-3)Man | 0.057±0.004 | 0.072±0.003 | 0.081±0.011 | | 0.079±0.005 | 0.093±0.011 | 1.25 | 1.4 | 1.37 | 1.62*** |
| MAL-I | | Galβ-1,4GlcNAc, Siaα2-3Gal, Galβ1-3GlcNAc, Siaα2-3 | 0.001±0.002 | 0.015±0.002 | 0.010±0.007 | | 0.013±0.001 | 0.010±0.000 | 12.91*** | 8.79*** | 11.59*** | 9.18*** |
| BPL | | Galβ1-3GalNAc, terminal GalNAc | 0.006±0.000 | 0.016±0.001 | 0.015±0.000 | | 0.017±0.002 | 0.012±0.000 | 2.8*** | 3.04*** | 2.65*** | 2.08*** |
| **Decreased lectin signals** | | | | | | | | | | | | |
| ECA | Galβ-1,4GlcNAc (type II), Galβ1-3GlcNAc (type I) | | 0.017±0.002 | 0.015±0.005 | 0.017±0.001 | | 0.014±0.002 | 0.008±0.006 | 0.87 | 0.98 | 0.84 | 0.44* |
| MAL-II | Siaα2-3Galβ1-4Glc (NAc)/Glc | | 0.024±0.001 | 0.017±0.001 | 0.015±0.001 | | 0.017±0.001 | 0.015±0.001 | 0.73 | 0.64*** | 0.7 | 0.62*** |
| PTL-I | GalNAc, GalNAcα-1,3Gal, GalNAcα-1,3Galβ-1,3/4Glc | | 0.024±0.001 | 0.019±0.001 | 0.017±0.002 | | 0.018±0.000 | 0.014±0.002 | 0.8 | 0.7 | 0.76 | 0.58*** |
| SJA | Terminal in GalNAc and Gal | | 0.021±0.001 | 0.018±0.000 | 0.017±0.002 | | 0.017±0.001 | 0.014±0.002 | 0.86 | 0.81 | 0.79 | 0.65*** |
| AAL | Fucα1-6 GlcNAc(core fucose), Fucα1-3(Galβ1-4)GlcNAc | | 0.035±0.001 | 0.031±0.001 | 0.021±0.014 | | 0.030±0.001 | 0.028±0.002 | 0.86 | 0.59* | 0.84 | 0.78 |
| LTL | Fucα1-3(Galβ1-4)GlcNAc | | 0.033±0.001 | 0.000±0.000 | 0.016±0.011 | | 0.007±0.012 | 0.007±0.006 | 0*** | 0.48* | 0.21** | 0.2** |
| MPL | Galβ1-3GalNAc, GalNAc | | 0.023±0.001 | 0.018±0.001 | 0.016±0.001 | | 0.016±0.001 | 0.016±0.001 | 0.77 | 0.7 | 0.68 | 0.66*** |
| DBA | GalNAcα1-3(Fucα1-2)Gal (blood group A antigen),αGalNAc, Tn antigen | | 0.023±0.002 | 0.000±0.000 | 0.004±0.009 | | 0.005±0.008 | 0.001±0.002 | 0*** | 0.18** | 0.19** | 0.03** |
| RCA120 | β-Gal, Galβ-1,4GlcNAc (type II), Galβ1-3GlcNAc (type I) | | 0.034±0.003 | 0.032±0.003 | 0.033±0.004 | | 0.024±0.000 | 0.022±0.001 | 0.94 | 0.95 | 0.69 | 0.63*** |
| STL | Core (GlcNAc) of N-glycan | | 0.047±0.007 | 0.030±0.002 | 0.031±0.003 | | 0.032±0.003 | 0.027±0.002 | 0.63*** | 0.66*** | 0.67 | 0.57*** |
| PTL-II | Gal, blood group H, T-antigen | | 0.026±0.002 | 0.020±0.001 | 0.019±0.001 | | 0.010±0.009 | 0.017±0.003 | 0.76 | 0.71 | 0.35*** | 0.62** |
| DSA | β-D-GlcNA, (GlcNAcβ1-4)n, Galβ1-4GlcNAc | | 0.029±0.001 | 0.020±0.001 | 0.019±0.001 | | 0.018±0.001 | 0.019±0.001 | 0.67 | 0.64*** | 0.62*** | 0.63*** |
| PSA | α-D-Man, Fucα-1,6GlcNAc, α-D-Glc | | 0.034±0.006 | 0.013±0.011 | 0.012±0.004 | | 0.022±0.001 | 0.008±0.006 | 0.36** | 0.57* | 0.65* | 0.24** |
| UEA-I | Fucα1-2Galβ1-4Glc(NAc) | | 0.020±0.004 | 0.002±0.003 | 0.003±0.003 | | 0.005±0.002 | 0.006±0.001 | 0.09*** | 0.13*** | 0.25*** | 0.27*** |
| PHA-E+L | Bisecting GlcNAc, bi-antennary N-glycans,tri- and tetra-antennary complex-type N-glycan | | 0.007±0.001 | 0.000±0.000 | 0.000±0.000 | | 0.002±0.002 | 0.000±0.000 | 0*** | 0*** | 0.28*** | 0*** |

Note: Among these 37 lectins, signal from 6, 15 and 16 kinds of lectins were found to be increased (ratio (1-7 dpa/0 dpa) > 1.5), decreased (ratio (1-7 dpa/0 dpa) < 0.67), or unchanged during the regeneration process. The 16 unchanged lectins are not displayed in this table.

**Table S3** Quantification of the identified core fucose-binding glycoproteins (CFBGs) during head regeneration of planarians using the LC-MS/MS.

| **Protein Name** | **0 dpa** | **1 dpa** | **3 dpa** | **5 dpa** | **7 dpa** |
| --- | --- | --- | --- | --- | --- |
| DjpiwiB | + | + | + | + | + |
| DjpiwiA | + | + | + | + | + |
| Flotillin-1 | + | + | + | + | + |
| Flotillin-2 | + | + | + | + | + |
| Peroxiredoxin-3 | + | + | + | + | + |
| Trypsin-like serine protease | + | + | + | + | + |
| Elongation factor 1-alpha | + | + | + | + | + |
| Tubulin alpha chain | + | + | + | + | + |
| Myosin heavy chain | + | + | + | + | + |
| Small HSP protein | + | + | + | + | + |
| Innexin | + | + | + | + | + |
| Myosin heavy chain | + | + | + | + | + |
| Glutathione S-transferase | + | + | + | + | + |
| ADP-ribosylation factor | + | + | + | + | + |
| Glyceraldehyde-3-phosphate dehydrogenase | + | + | + | + | + |
| Beta-actin | + | + | + | + | + |
| Aquaporin | + | + | + | + | + |
| Glucose-regulated protein 78 | + | + | + | + | + |
| Heat shock protein 70 | + | + | + | + | + |
| Puromycin-sensitive aminopeptidase | + | + | + | + | + |
| Thioredoxin glutathione reductase | + | + |  |  |  |
| Inositol 1,4,5-trisphosphate receptor | + | + |  |  |  |
| Tyramine beta-hydroxylase | + | + |  |  |  |
| DEAD box polypeptide 48 protein (Fragment) | + | + |  | + |  |
| HnRNP F protein (Fragment) | + | + |  | + |  |
| Peroxiredoxin-1 | + | + |  | + | + |
| Contactin/TAG-1 cell adhesion molecule | + | + | + |  | + |
| Mortalin-like protein | + | + | + |  | + |
| Clathrin heavy chain | + | + | + |  |  |
| WM6 protein | + | + | + | + |  |
| Prohibitin 2 | + | + | + | + |  |
| EIF4A isoform 1A protein | + | + | + | + |  |
| Protein Wnt (Fragment) | + |  |  |  |  |
| Glutathione synthetase | + |  |  |  |  |
| PRP2 protein (Fragment) |  | + |  |  | + |
| DjVLGB |  | + | + |  |  |
| Sodium/potassium-transporting ATPase subunit alpha |  | + | + | + |  |
| Myosin light chain protein |  | + | + | + | + |
| Tryptophan hydroxylase |  | + |  | + |  |
| Intermediate filament protein |  |  |  | + | + |
| DjVLGA |  | + |  |  |  |
| Voltage-operated calcium channel subunitβ-1 |  | + |  |  |  |
| 90 kDa heat shock protein |  | + |  |  |  |
| Exoribonuclease-1 (Fragment) |  | + |  |  |  |
| L1-like cell adhesion molecule |  | + |  |  |  |
| Innexin 8 |  |  | + |  |  |
| Innexin 9 |  |  | + |  |  |
| Eukaryotic initiation factor-4A3 |  |  | + |  |  |
| Cell adhesion molecule |  |  | + |  |  |
| Kinesin-like protein |  |  |  | + |  |
| Uncoordinated 5 |  |  |  |  | + |
| Intermediate filament b |  |  |  |  | + |
| Winged helix/forkhead transcription factor DjFoxA |  |  |  |  | + |

Note: + represents that the protein was identified at the specific regenerative time points.

**Table S4** Detailed information about CFBGs analyzed by GO Ontology.

| **Class** | | **Protein ID** | | **Protein** |
| --- | --- | --- | --- | --- |
| **Biological process** | | | | |
| Biological regulation | tr\|M9NUP0\|M9NUP0_DUGJA | | Peroxiredoxin-3 | |
|  | tr\|D2Z0D8\|D2Z0D8_DUGJA | | DjPIWIA | |
|  | tr\|D2Z0D9\|D2Z0D9_DUGJA | | DjPIWIB | |
|  | sp\|P91924\|ARF_DUGJA | | ADP-ribosylation factor | |
| cellular component organization or biogenesis | tr\|F8WQS7\|F8WQS7_DUGJA | | Tubulin alpha chain | |
| cellular process | r\|A4V6L7\|A4V6L7_DUGJA | | DEAD box polypeptide 48 protein; | |
|  | tr\|F8WQS7\|F8WQS7_DUGJA | | Tubulin alpha chain | |
|  | tr\|A4V6M7\|A4V6M7_DUGJA | | WM6 protein | |
|  | tr\|C0KTP7\|C0KTP7_DUGJA | | 90 kDa heat shock protein | |
|  | tr\|D1GJ91\|D1GJ91_DUGJA | | Mortalin-like protein | |
|  | tr\|Q23959\|Q23959_DUGJA | | Elongation factor 1-alpha | |
|  | tr\|M9NUP0\|M9NUP0_DUGJA | | Peroxiredoxin-3 | |
|  | tr\|D2Z0D8\|D2Z0D8_DUGJA | | DjPIWIA | |
|  | tr\|D2Z0D9\|D2Z0D9_DUGJA | | DjPIWIB | |
|  | sp\|P91924\|ARF_DUGJA | | ADP-ribosylation factor | |
|  | tr\|O76154\|O76154_DUGJA | | Sodium/potassium-transporting ATPase subunit alpha | |
|  | tr\|D5JG53\|D5JG53_DUGJA | | Eukaryotic initiation factor-4A3 | |
|  | tr\|Q9NDK9\|Q9NDK9_DUGJA | | Winged helix/forkhead transcription factor DjFoxA | |
| developmental process | sp\|P91924\|ARF_DUGJA | | ADP-ribosylation factor | |
| localization | tr\|A4V6M7\|A4V6M7_DUGJA | | WM6 protein | |
|  | tr\|O76154\|O76154_DUGJA | | Sodium/potassium-transporting ATPase subunit alpha | |
|  | tr\|B8Y5Z1\|B8Y5Z1_DUGJA | | Voltage-operated calcium channel subunit beta-1 | |
|  | sp\|P91924\|ARF_DUGJA | | ADP-ribosylation factor | |
| metabolic process | tr\|F8WQS7\|F8WQS7_DUGJA | | Tubulin alpha chain | |
|  | tr\|A4V6M7\|A4V6M7_DUGJA | | WM6 protein | |
|  | tr\|C0KTP7\|C0KTP7_DUGJA | | 90 kDa heat shock protein | |
|  | tr\|D1GJ91\|D1GJ91_DUGJA | | Mortalin-like protein | |
|  | tr\|A4V6L7\|A4V6L7_DUGJA | | DEAD box polypeptide 48 protein | |
|  | tr\|Q23959\|Q23959_DUGJA | | Elongation factor 1-alpha | |
|  | tr\|D2Z0D8\|D2Z0D8_DUGJA | | DjPIWIA | |
|  | tr\|D2Z0D9\|D2Z0D9_DUGJA | | DjPIWIB | |
|  | tr\|D0VYP9\|D0VYP9_DUGJA | | Glyceraldehyde-3-phosphate dehydrogenase | |
|  | tr\|O76154\|O76154_DUGJA | | Sodium/potassium-transporting ATPase subunit alpha | |
|  | tr\|D5JG53\|D5JG53_DUGJA | | Eukaryotic initiation factor-4A3 | |
|  | tr\|Q9NDK9\|Q9NDK9_DUGJA | | Winged helix/forkhead transcription factor DjFoxA | |
| multi-organism process | sp\|P91924\|ARF_DUGJA | | ADP-ribosylation factor | |
| multicellular organismal process | sp\|P91924\|ARF_DUGJA | | ADP-ribosylation factor | |
|  | tr\|D2Z0D9\|D2Z0D9_DUGJA | | DjPIWIB | |
| negative regulation of biological process | tr\|D2Z0D8\|D2Z0D8_DUGJA | | DjPIWIA | |
|  | tr\|D2Z0D9\|D2Z0D9_DUGJA | | DjPIWIB | |
| regulation of biological process | tr\|D2Z0D8\|D2Z0D8_DUGJA | | DjPIWIA | |
|  | tr\|D2Z0D9\|D2Z0D9_DUGJA | | DjPIWIB | |
|  | sp\|P91924\|ARF_DUGJA | | ADP-ribosylation factor | |
| reproduction | sp\|P91924\|ARF_DUGJA | | ADP-ribosylation factor | |
| reproductive process | sp\|P91924\|ARF_DUGJA | | ADP-ribosylation factor | |
| response to stimulus | tr\|A4V6L7\|A4V6L7_DUGJA | | DEAD box polypeptide 48 protein | |
|  | tr\|C0KTP7\|C0KTP7_DUGJA | | 90 kDa heat shock protein | |
|  | tr\|B0LVF7\|B0LVF7_DUGJA | | Heat shock protein 70 | |
|  | tr\|G8JF14\|G8JF14_DUGJA | | Glucose-regulated protein 78 | |
|  | sp\|P91924\|ARF_DUGJA | | ADP-ribosylation factor | |
|  | tr\|D5JG53\|D5JG53_DUGJA | | Eukaryotic initiation factor-4A3 | |
| signaling | sp\|P91924\|ARF_DUGJA | | ADP-ribosylation factor | |
| single-organism process | sp\|P91924\|ARF_DUGJA; | | ADP-ribosylation factor | |
|  | tr\|F8WQS7\|F8WQS7_DUGJA | | Tubulin alpha chain | |
|  | tr\|M9NUP0\|M9NUP0_DUGJA | | Peroxiredoxin-3 | |
|  | tr\|D2Z0D8\|D2Z0D8_DUGJA | | DjPIWIA | |
|  | tr\|D2Z0D9\|D2Z0D9_DUGJA | | DjPIWIB | |
|  | tr\|D0VYP9\|D0VYP9_DUGJA | | Glyceraldehyde-3-phosphate dehydrogenase | |
|  | tr\|O76154\|O76154_DUGJA | | Sodium/potassium-transporting ATPase subunit alpha | |
|  | tr\|B8Y5Z1\|B8Y5Z1_DUGJA | | Voltage-operated calcium channel subunit beta-1 | |
|  | tr\|A4V6M7\|A4V6M7_DUGJA | | WM6 protein | |
|  | tr\|Q23959\|Q23959_DUGJA | | Elongation factor 1-alpha | |
| **Cellular component** | | | | |
| cell | sp\|P91924\|ARF_DUGJA | | ADP-ribosylation factor | |
|  | tr\|C0KTP7\|C0KTP7_DUGJA | | 90 kDa heat shock protein | |
|  | tr\|D0VYP9\|D0VYP9_DUGJA | | Glyceraldehyde-3-phosphate dehydrogenase | |
|  | tr\|H2DRI7\|H2DRI7_DUGJA | | Trypsin-like serine protease | |
|  | tr\|Q23959\|Q23959_DUGJA | | Elongation factor 1-alpha | |
|  | tr\|A0A0A7AD70\|A0A0A7AD70_DUGJA | | Prohibitin 2 | |
|  | tr\|K7X7R0\|K7X7R0_DUGJA | | Beta-actin | |
|  | tr\|A4V6L7\|A4V6L7_DUGJA | | DEAD box polypeptide 48 protein | |
|  | tr\|G8JF14\|G8JF14_DUGJA | | Glucose-regulated protein 78 | |
|  | tr\|F8WQS7\|F8WQS7_DUGJA | | Tubulin alpha chain | |
|  | tr\|D5JG53\|D5JG53_DUGJA | | Eukaryotic initiation factor-4A3 | |
|  | tr\|Q9NDK9\|Q9NDK9_DUGJA | | Winged helix/forkhead transcription factor DjFoxA | |
|  | tr\|A4V6M7\|A4V6M7_DUGJA | | WM6 protein | |
|  | tr\|O96062\|O96062_DUGJA | | Myosin heavy chain | |
| cell part | sp\|P91924\|ARF_DUGJA | | ADP-ribosylation factor | |
|  | tr\|A4V6M7\|A4V6M7_DUGJA | | WM6 protein | |
|  | tr\|O96062\|O96062_DUGJA | | Myosin heavy chain | |
|  | tr\|C0KTP7\|C0KTP7_DUGJA | | 90 kDa heat shock protein | |
|  | tr\|D0VYP9\|D0VYP9_DUGJA | | Glyceraldehyde-3-phosphate dehydrogenase | |
|  | tr\|H2DRI7\|H2DRI7_DUGJA | | Trypsin-like serine protease | |
|  | tr\|Q23959\|Q23959_DUGJA | | Elongation factor 1-alpha | |
|  | tr\|A0A0A7AD70\|A0A0A7AD70_DUGJA | | Prohibitin 2 | |
|  | tr\|K7X7R0\|K7X7R0_DUGJA | | Beta-actin | |
|  | tr\|A4V6L7\|A4V6L7_DUGJA | | DEAD box polypeptide 48 protein | |
|  | tr\|F8WQS7\|F8WQS7_DUGJA | | Tubulin alpha chain | |
|  | tr\|G8JF14\|G8JF14_DUGJA | | Glucose-regulated protein 78 | |
|  | tr\|D5JG53\|D5JG53_DUGJA | | Eukaryotic initiation factor-4A3 | |
|  | tr\|Q9NDK9\|Q9NDK9_DUGJA | | Winged helix/forkhead transcription factor DjFoxA | |
| macromolecular complex | tr\|A4V6L7\|A4V6L7_DUGJA | | DEAD box polypeptide 48 protein | |
|  | tr\|A4V6M7\|A4V6M7_DUGJA | | WM6 protein | |
|  | tr\|F8WQS7\|F8WQS7_DUGJA | | Tubulin alpha chain | |
|  | tr\|O96062\|O96062_DUGJA | | Myosin heavy chain | |
|  | tr\|D5JG53\|D5JG53_DUGJA | | Eukaryotic initiation factor-4A3 | |
|  | tr\|Q95P01\|Q95P01_DUGJA | | Intermediate filament b | |
| membrane | tr\|O76154\|O76154_DUGJA | | Sodium/potassium-transporting ATPase subunit alpha; | |
| membrane part | tr\|O76154\|O76154_DUGJA | | Sodium/potassium-transporting ATPase subunit alpha; | |
| membrane-enclosed lumen | tr\|A4V6M7\|A4V6M7_DUGJA | | WM6 protein | |
| organelle | sp\|P91924\|ARF_DUGJA | | ADP-ribosylation factor | |
|  | tr\|A4V6M7\|A4V6M7_DUGJA | | WM6 protein | |
|  | tr\|K7X7R0\|K7X7R0_DUGJA | | Beta-actin | |
|  | tr\|A4V6L7\|A4V6L7_DUGJA | | DEAD box polypeptide 48 protein | |
|  | tr\|F8WQS7\|F8WQS7_DUGJA | | Tubulin alpha chain | |
|  | tr\|G8JF14\|G8JF14_DUGJA | | Glucose-regulated protein 78 | |
|  | tr\|O96062\|O96062_DUGJA | | Myosin heavy chain | |
|  | tr\|D5JG53\|D5JG53_DUGJA | | Eukaryotic initiation factor-4A3 | |
|  | tr\|Q9NDK9\|Q9NDK9_DUGJA | | Winged helix/forkhead transcription factor DjFoxA | |
| organelle part | tr\|A4V6M7\|A4V6M7_DUGJA | | WM6 protein | |
|  | tr\|A4V6L7\|A4V6L7_DUGJA | | DEAD box polypeptide 48 protein | |
|  | tr\|G8JF14\|G8JF14_DUGJA | | Glucose-regulated protein 78 | |
|  | tr\|O96062\|O96062_DUGJA | | Myosin heavy chain | |
|  | tr\|D5JG53\|D5JG53_DUGJA | | Eukaryotic initiation factor-4A3 | |
| **Molecular function** | | | | |
| antioxidant activity | tr\|M9NTZ9\|M9NTZ9_DUGJA | | Peroxiredoxin-1 | |
|  | tr\|M9NUP0\|M9NUP0_DUGJA | | Peroxiredoxin-3 | |
| binding | tr\|D2Z0D8\|D2Z0D8_DUGJA | | DjPIWIA | |
|  | tr\|D2Z0D9\|D2Z0D9_DUGJA | | DjPIWIB | |
|  | tr\|A4V6M7\|A4V6M7_DUGJA | | WM6 protein | |
|  | tr\|C0KTP7\|C0KTP7_DUGJA | | 90 kDa heat shock protein | |
|  | tr\|D1GJ91\|D1GJ91_DUGJA | | Mortalin-like protein | |
|  | tr\|O96062\|O96062_DUGJA | | Myosin heavy chain | |
|  | tr\|A4V6L8\|A4V6L8_DUGJA | | PRP2 protein | |
|  | tr\|D0VYP9\|D0VYP9_DUGJA | | Glyceraldehyde-3-phosphate dehydrogenase | |
|  | tr\|O97032\|O97032_DUGJA | | DjVLGB | |
|  | tr\|A4V6L7\|A4V6L7_DUGJA | | DEAD box polypeptide 48 protein | |
|  | tr\|B0LVF7\|B0LVF7_DUGJA | | Heat shock protein 70 | |
|  | tr\|G8JF14\|G8JF14_DUGJA | | Glucose-regulated protein 78 | |
|  | tr\|K7X7R0\|K7X7R0_DUGJA | | Beta-actin | |
|  | tr\|O76154\|O76154_DUGJA | | Sodium/potassium-transporting ATPase subunit alpha; | |
|  | tr\|Q23959\|Q23959_DUGJA | | Elongation factor 1-alpha | |
|  | tr\|E5Q336\|E5Q336_DUGJA | | Myosin light chain protein | |
|  | tr\|A0A068AVN2\|A0A068AVN2_DUGJA | | Thioredoxin glutathione reductase | |
|  | sp\|P91924\|ARF_DUGJA | | ADP-ribosylation factor | |
|  | tr\|F8WQS7\|F8WQS7_DUGJA | | Tubulin alpha chain | |
|  | tr\|M9NTD0\|M9NTD0_DUGJA | | Glutathione synthetase | |
|  | tr\|D5JG53\|D5JG53_DUGJA | | Eukaryotic initiation factor-4A3 | |
|  | tr\|Q9NDK9\|Q9NDK9_DUGJA | | Winged helix/forkhead transcription factor DjFoxA | |
| catalytic activity | tr\|A4V6L7\|A4V6L7_DUGJA | | DEAD box polypeptide 48 protein | |
|  | tr\|A4V6M7\|A4V6M7_DUGJA | | WM6 protein | |
|  | tr\|H2DRI7\|H2DRI7_DUGJA | | Trypsin-like serine protease | |
|  | tr\|A4V6L8\|A4V6L8_DUGJA | | PRP2 protein | |
|  | tr\|F8WQS7\|F8WQS7_DUGJA | | Tubulin alpha chain | |
|  | tr\|O97032\|O97032_DUGJA | | DjVLGB | |
|  | tr\|Q23959\|Q23959_DUGJA | | Elongation factor 1-alpha | |
|  | tr\|A0A068AVN2\|A0A068AVN2_DUGJA | | Thioredoxin glutathione reductase | |
|  | tr\|M9NUP0\|M9NUP0_DUGJA | | Peroxiredoxin-3 | |
|  | tr\|D1GJ91\|D1GJ91_DUGJA | | Mortalin-like protein | |
|  | tr\|M9NTZ9\|M9NTZ9_DUGJA | | Peroxiredoxin-1 | |
|  | tr\|O76154\|O76154_DUGJA | | Sodium/potassium-transporting ATPase subunit alpha; | |
|  | tr\|D5JG53\|D5JG53_DUGJA | | Eukaryotic initiation factor-4A3 | |
| transporter activity | tr\|O76154\|O76154_DUGJA | | Sodium/potassium-transporting ATPase subunit alpha | |
| nucleic acid binding transcription factor activity | tr\|Q9NDK9\|Q9NDK9_DUGJA | | Winged helix/forkhead transcription factor DjFoxA | |

**Table S5** KEGG pathway enrichment analysis of differential expression proteins.

| **Pathway** | **Pathway ID** | **Proteins ID** |
| --- | --- | --- |
| Dorso-ventral axis formation | ko04320 | tr\|D2Z0D8\|D2Z0D8_DUGJA  tr\|D2Z0D9\|D2Z0D9_DUGJA |
| Cell adhesion molecules (CAMs) | ko04514 | tr\|Q1JUB3\|Q1JUB3_DUGJA  tr\|Q1JUB4\|Q1JUB4_DUGJA  tr\|Q1JUB6\|Q1JUB6_DUGJA |
| Basal transcription factors | ko03022 | tr\|Q2L6M6\|Q2L6M6_DUGJA  tr\|Q2L6M7\|Q2L6M7_DUGJA  tr\|Q2L6M8\|Q2L6M8_DUGJA |
| Hippo signaling pathway | ko04390 | tr\|K7X7R0\|K7X7R0_DUGJA  tr\|D0VYP8\|D0VYP8_DUGJA |
| Signaling pathways regulating pluripotency of stem cells | ko04550 | tr\|D0VYP8\|D0VYP8_DUGJA |
| Wnt signaling pathway | ko04310 | tr\|D0VYP8\|D0VYP8_DUGJA |
| HTLV-I infection | ko05166 | tr\|D0VYP8\|D0VYP8_DUGJA |
| Hedgehog signaling pathway | ko04340 | tr\|D0VYP8\|D0VYP8_DUGJA |
| Basal cell carcinoma | ko05217 | tr\|D0VYP8\|D0VYP8_DUGJA |
| Calcium signaling pathway | ko04020 | tr\|E5Q336\|E5Q336_DUGJA  tr\|R9S1J3\|R9S1J3_DUGJA |
| Protein processing in endoplasmic reticulum | ko04141 | tr\|B0LVF7\|B0LVF7_DUGJA  tr\|C0KTP7\|C0KTP7_DUGJA  tr\|G8JF14\|G8JF14_DUGJA  tr\|H1ZWN3\|H1ZWN3_DUGJA |
| cGMP-PKG signaling pathway | ko04022 | tr\|E5Q336\|E5Q336_DUGJA  tr\|O76154\|O76154_DUGJA  tr\|O96062\|O96062_DUGJA  tr\|O96063\|O96063_DUGJA  tr\|R9S1J3\|R9S1J3_DUGJA |
| RIG-I-like receptor signaling pathway | ko04622 | tr\|O97031\|O97031_DUGJA  tr\|O97032\|O97032_DUGJA |
| RNA transport | ko03013 | tr\|A4V6L7\|A4V6L7_DUGJA  tr\|A4V6M7\|A4V6M7_DUGJA  tr\|A4V6N0\|A4V6N0_DUGJA  tr\|Q23959\|Q23959_DUGJA  tr\|D5JG53\|D5JG53_DUGJA |
| Regulation of actin cytoskeleton | ko04810 | tr\|K7X7R0\|K7X7R0_DUGJA  tr\|O96062\|O96062_DUGJA  tr\|O96063\|O96063_DUGJA |
| Tight junction | ko04530 | tr\|K7X7R0\|K7X7R0_DUGJA  tr\|O96062\|O96062_DUGJA  tr\|O96063\|O96063_DUGJA |
| Rap1 signaling pathway | ko04015 | tr\|E5Q336\|E5Q336_DUGJA  tr\|K7X7R0\|K7X7R0_DUGJA |
| cAMP signaling pathway | ko04024 | tr\|E5Q336\|E5Q336_DUGJA  tr\|O76154\|O76154_DUGJA |
| Gap junction | ko04540 | tr\|F8WQS7\|F8WQS7_DUGJA  tr\|R9S1J3\|R9S1J3_DUGJA |
| MAPK signaling pathway | ko04010 | tr\|B0LVF7\|B0LVF7_DUGJA  tr\|B8Y5Z1\|B8Y5Z1_DUGJA |
| Proteoglycans in cancer | ko05205 | tr\|K7X7R0\|K7X7R0_DUGJA  tr\|R9S1J3\|R9S1J3_DUGJA  tr\|D0VYP8\|D0VYP8_DUGJA |
| Adherens junction | ko04520 | tr\|K7X7R0\|K7X7R0_DUGJA |
| Cysteine and methionine metabolism | ko00270 | tr\|M9NTD0\|M9NTD0_DUGJA |
| Tyrosine metabolism | ko00350 | tr\|B6ZH61\|B6ZH61_DUGJA |
| Leukocyte transendothelial migration | ko04670 | tr\|K7X7R0\|K7X7R0_DUGJA |
| Neurotrophin signaling pathway | ko04722 | tr\|E5Q336\|E5Q336_DUGJA |
| PI3K-Akt signaling pathway | ko04151 | tr\|C0KTP7\|C0KTP7_DUGJA |
| Tryptophan metabolism | ko00380 | tr\|A7VKD9\|A7VKD9_DUGJA |
| Maturity onset diabetes of the young | ko04950 | tr\|Q9NDK9\|Q9NDK9_DUGJA |
| Oxytocin signaling pathway | ko04921 | tr\|B8Y5Z1\|B8Y5Z1_DUGJA  tr\|E5Q336\|E5Q336_DUGJA  tr\|K7X7R0\|K7X7R0_DUGJA  tr\|R9S1J3\|R9S1J3_DUGJA |
| Vascular smooth muscle contraction | ko04270 | tr\|E5Q336\|E5Q336_DUGJA  tr\|O96062\|O96062_DUGJA  tr\|O96063\|O96063_DUGJA  tr\|R9S1J3\|R9S1J3_DUGJA |
| Legionellosis | ko05134 | sp\|P91924\|ARF_DUGJA  tr\|B0LVF7\|B0LVF7_DUGJA  tr\|Q23959\|Q23959_DUGJA |
| MAPK signaling pathway | ko04010 | tr\|B0LVF7\|B0LVF7_DUGJA  tr\|B8Y5Z1\|B8Y5Z1_DUGJA |
| Proteoglycans in cancer | ko05205 | tr\|K7X7R0\|K7X7R0_DUGJA  tr\|R9S1J3\|R9S1J3_DUGJA  tr\|D0VYP8\|D0VYP8_DUGJA |
| Adherens junction | ko04520 | tr\|K7X7R0\|K7X7R0_DUGJA |
| Cysteine and methionine metabolism | ko00270 | tr\|M9NTD0\|M9NTD0_DUGJA |
| Tyrosine metabolism | ko00350 | tr\|B6ZH61\|B6ZH61_DUGJA |
| Leukocyte transendothelial migration | ko04670 | tr\|K7X7R0\|K7X7R0_DUGJA |
| Neurotrophin signaling pathway | ko04722 | tr\|E5Q336\|E5Q336_DUGJA |
| PI3K-Akt signaling pathway | ko04151 | tr\|C0KTP7\|C0KTP7_DUGJA |
| Tryptophan metabolism | ko00380 | tr\|A7VKD9\|A7VKD9_DUGJA |
| Ras signaling pathway | ko04014 | tr\|E5Q336\|E5Q336_DUGJA |
| Focal adhesion | ko04510 | tr\|K7X7R0\|K7X7R0_DUGJA |
| Retrograde endocannabinoid signaling | ko04723 | tr\|R9S1J3\|R9S1J3_DUGJA |
| Endocytosis | ko04144 | sp\|P91924\|ARF_DUGJA  tr\|A5HUF0\|A5HUF0_DUGJA  tr\|B0LVF7\|B0LVF7_DUGJA |
| Antigen processing and presentation | ko04612 | tr\|B0LVF7\|B0LVF7_DUGJA;  tr\|C0KTP7\|C0KTP7_DUGJA;  tr\|G8JF14\|G8JF14_DUGJA |
| Adrenergic signaling in cardiomyocytes | ko04261 | tr\|B8Y5Z1\|B8Y5Z1_DUGJA  tr\|E5Q336\|E5Q336_DUGJA  tr\|O76154\|O76154_DUGJA  tr\|O96062\|O96062_DUGJA  tr\|O96063\|O96063_DUGJA |
| Maturity onset diabetes of the young | ko04950 | tr\|Q9NDK9\|Q9NDK9_DUGJA |
| Oxytocin signaling pathway | ko04921 | tr\|B8Y5Z1\|B8Y5Z1_DUGJA  tr\|E5Q336\|E5Q336_DUGJA  tr\|K7X7R0\|K7X7R0_DUGJA  tr\|R9S1J3\|R9S1J3_DUGJA |
| Vascular smooth muscle contraction | ko04270 | tr\|E5Q336\|E5Q336_DUGJA  tr\|O96062\|O96062_DUGJA  tr\|O96063\|O96063_DUGJA  tr\|R9S1J3\|R9S1J3_DUGJA |
| Spliceosome | ko03040 | tr\|A4V6L7\|A4V6L7_DUGJA  tr\|A4V6L8\|A4V6L8_DUGJA  tr\|A4V6M7\|A4V6M7_DUGJA  tr\|B0LVF7\|B0LVF7_DUGJA  tr\|D5JG53\|D5JG53_DUGJA |
| Dilated cardiomyopathy | ko05414 | tr\|B8Y5Z1\|B8Y5Z1_DUGJA  tr\|K7X7R0\|K7X7R0_DUGJA  tr\|O96062\|O96062_DUGJA  tr\|O96063\|O96063_DUGJA |
| Cardiac muscle contraction | ko04260 | tr\|B8Y5Z1\|B8Y5Z1_DUGJA;  tr\|O76154\|O76154_DUGJA  tr\|O96062\|O96062_DUGJA  tr\|O96063\|O96063_DUGJA |
| Thyroid hormone signaling pathway | ko04919 | tr\|K7X7R0\|K7X7R0_DUGJA  tr\|O76154\|O76154_DUGJA  tr\|O96062\|O96062_DUGJA  tr\|O96063\|O96063_DUGJA  tr\|D0VYP8\|D0VYP8_DUGJA |
| Gastric acid secretion | ko04971 | tr\|E5Q336\|E5Q336_DUGJA  tr\|K7X7R0\|K7X7R0_DUGJA  tr\|O76154\|O76154_DUGJA  tr\|R9S1J3\|R9S1J3_DUGJA |
| Hypertrophic cardiomyopathy (HCM) | ko05410 | tr\|B8Y5Z1\|B8Y5Z1_DUGJA  tr\|K7X7R0\|K7X7R0_DUGJA  tr\|O96062\|O96062_DUGJA  tr\|O96063\|O96063_DUGJA |
| Estrogen signaling pathway | ko04915 | tr\|B0LVF7\|B0LVF7_DUGJA  tr\|C0KTP7\|C0KTP7_DUGJA  tr\|E5Q336\|E5Q336_DUGJA  tr\|R9S1J3\|R9S1J3_DUGJA |
| Viral myocarditis | ko05416 | tr\|K7X7R0\|K7X7R0_DUGJA  tr\|O96062\|O96062_DUGJA  tr\|O96063\|O96063_DUGJA |
| Insulin signaling pathwa | ko04910 | tr\|A0A068CL83\|A0A068CL83_DUGJA  tr\|A0A068CQN6\|A0A068CQN6_DUGJA  tr\|E5Q336\|E5Q336_DUGJA |
| Salivary secretion | ko04970 | tr\|E5Q336\|E5Q336_DUGJA  tr\|O76154\|O76154_DUGJA  tr\|R9S1J3\|R9S1J3_DUGJA |
| Legionellosis | ko05134 | sp\|P91924\|ARF_DUGJA  tr\|B0LVF7\|B0LVF7_DUGJA  tr\|Q23959\|Q23959_DUGJA |
| Influenza A | ko05164 | tr\|A4V6M7\|A4V6M7_DUGJA  tr\|B0LVF7\|B0LVF7_DUGJA  tr\|K7X7R0\|K7X7R0_DUGJA |
| Metabolic pathways | ko01100 | tr\|A7VKD9\|A7VKD9_DUGJA  tr\|B6ZH61\|B6ZH61_DUGJA  tr\|D0VYP9\|D0VYP9_DUGJA  tr\|M9NTD0\|M9NTD0_DUGJA |
| Alzheimer's disease | ko05010 | tr\|D0VYP9\|D0VYP9_DUGJA  tr\|E5Q336\|E5Q336_DUGJA  tr\|R9S1J3\|R9S1J3_DUGJA |
| Thyroid hormone synthesis | ko04918 | tr\|G8JF14\|G8JF14_DUGJA  tr\|O76154\|O76154_DUGJA  tr\|R9S1J3\|R9S1J3_DUGJA |
| Phototransduction - fly | ko04745 | tr\|E5Q336\|E5Q336_DUGJA  tr\|K7X7R0\|K7X7R0_DUGJA  tr\|R9S1J3\|R9S1J3_DUGJA |
| Salmonella infection | ko05132 | tr\|K7X7R0\|K7X7R0_DUGJA  tr\|O96062\|O96062_DUGJA  tr\|O96063\|O96063_DUGJA |
| Circadian entrainment | ko04713 | tr\|E5Q336\|E5Q336_DUGJA  tr\|R9S1J3\|R9S1J3_DUGJA |
| Tuberculosis | ko05152 | tr\|D1GJ91\|D1GJ91_DUGJA  tr\|E5Q336\|E5Q336_DUGJA |
| Prostate cancer | ko05215 | tr\|C0KTP7\|C0KTP7_DUGJA  tr\|G9BRW2\|G9BRW2_DUGJA |
| GnRH signaling pathway | ko04912 | tr\|E5Q336\|E5Q336_DUGJA  tr\|R9S1J3\|R9S1J3_DUGJA |
| Bacterial invasion of epithelial cells | ko05100 | tr\|A5HUF0\|A5HUF0_DUGJA  tr\|K7X7R0\|K7X7R0_DUGJA |
| Viral carcinogenesis | ko05203 | tr\|O97031\|O97031_DUGJA  tr\|O97032\|O97032_DUGJA |
| Huntington's disease | ko05016 | tr\|A5HUF0\|A5HUF0_DUGJA  tr\|R9S1J3\|R9S1J3_DUGJA |
| Bile secretion | ko04976 | tr\|A0A0G4DCR1\|A0A0G4DCR1_DUGJA  tr\|O76154\|O76154_DUGJA |
| Platelet activation | ko04611 | tr\|K7X7R0\|K7X7R0_DUGJA  tr\|R9S1J3\|R9S1J3_DUGJA |
| Renin secretion | ko04924 | tr\|E5Q336\|E5Q336_DUGJA  tr\|R9S1J3\|R9S1J3_DUGJA |
| Serotonergic synapse | ko04726 | tr\|A7VKD9\|A7VKD9_DUGJA  tr\|R9S1J3\|R9S1J3_DUGJA |
| Hepatitis B | ko05161 | tr\|O97031\|O97031_DUGJA  tr\|O97032\|O97032_DUGJA |
| Endocrine and other factor-regulated calcium reabsorption | ko04961 | tr\|A5HUF0\|A5HUF0_DUGJA  tr\|O76154\|O76154_DUGJA |
| mRNA surveillance pathway | ko03015 | tr\|A4V6L7\|A4V6L7_DUGJA  tr\|A4V6M7\|A4V6M7_DUGJA  tr\|D5JG53\|D5JG53_DUGJA |
| Aldosterone synthesis and secretion | ko04925 | tr\|E5Q336\|E5Q336_DUGJA  tr\|R9S1J3\|R9S1J3_DUGJA |
| Arrhythmogenic right ventricular cardiomyopathy (ARVC) | ko05412 | tr\|B8Y5Z1\|B8Y5Z1_DUGJA  tr\|K7X7R0\|K7X7R0_DUGJA |
| Glucagon signaling pathway | ko04922 | tr\|E5Q336\|E5Q336_DUGJA  tr\|R9S1J3\|R9S1J3_DUGJA |
| Prion diseases | ko05020 | tr\|B0LVF7\|B0LVF7_DUGJA  tr\|G8JF14\|G8JF14_DUGJA  tr\|Q1JUB6\|Q1JUB6_DUGJA |
| Inflammatory mediator regulation of TRP channels | ko04750 | tr\|E5Q336\|E5Q336_DUGJA  tr\|R9S1J3\|R9S1J3_DUGJA |
| Phosphatidylinositol signaling system | ko04070 | tr\|E5Q336\|E5Q336_DUGJA  tr\|R9S1J3\|R9S1J3_DUGJA |
| Pathways in cancer | ko05200 | tr\|C0KTP7\|C0KTP7_DUGJA  tr\|G9BRW2\|G9BRW2_DUGJA  tr\|D0VYP8\|D0VYP8_DUGJA |
| Oocyte meiosis | ko04114 | tr\|E5Q336\|E5Q336_DUGJA  tr\|R9S1J3\|R9S1J3_DUGJA |
| Vibrio cholerae infection | ko05110 | sp\|P91924\|ARF_DUGJA  tr\|K7X7R0\|K7X7R0_DUGJA |
| Pathogenic Escherichia coli infection | ko05130 | tr\|F8WQS7\|F8WQS7_DUGJA  tr\|K7X7R0\|K7X7R0_DUGJA |
| Long-term potentiation | ko04720 | tr\|E5Q336\|E5Q336_DUGJA  tr\|R9S1J3\|R9S1J3_DUGJA |
| Pancreatic secretion | ko04972 | tr\|O76154\|O76154_DUGJA  tr\|R9S1J3\|R9S1J3_DUGJA |
| Phagosome | ko04145 | tr\|F8WQS7\|F8WQS7_DUGJA  tr\|K7X7R0\|K7X7R0_DUGJA |
| Dopaminergic synapse | ko04728 | tr\|E5Q336\|E5Q336_DUGJA  tr\|R9S1J3\|R9S1J3_DUGJA |
| RNA degradation | ko03018 | tr\|D1GJ91\|D1GJ91_DUGJA  tr\|D5JG38\|D5JG38_DUGJA |
| Aldosterone-regulated sodium reabsorption | ko04960 | tr\|O76154\|O76154_DUGJA |
| Peroxisome | ko04146 | tr\|M9NTZ9\|M9NTZ9_DUGJA |
| Biosynthesis of amino acids | ko01230 | tr\|D0VYP9\|D0VYP9_DUGJA |
| Hippo signaling pathway - fly | ko04391 | tr\|K7X7R0\|K7X7R0_DUGJA |
| Cholinergic synapse | ko04725 | tr\|R9S1J3\|R9S1J3_DUGJA |
| Mineral absorption | ko04978 | tr\|O76154\|O76154_DUGJA |
| Selenocompound metabolism | ko00450 | tr\|A0A068AVN2\|A0A068AVN2_DUGJA |
| Axon guidance | ko04360 | tr\|E1CHK7\|E1CHK7_DUGJA |
| Carbohydrate digestion and absorption | ko04973 | tr\|O76154\|O76154_DUGJA |
| Alcoholism | ko05034 | tr\|E5Q336\|E5Q336_DUGJA |
| Amphetamine addiction | ko05031 | tr\|E5Q336\|E5Q336_DUGJA |
| Insulin secretion | ko04911 | tr\|O76154\|O76154_DUGJA |
| Protein export | ko03060 | tr\|G8JF14\|G8JF14_DUGJA |
| Melanogenesis | ko04916 | tr\|E5Q336\|E5Q336_DUGJA  tr\|D0VYP8\|D0VYP8_DUGJA |
| Chemical carcinogenesis | ko05204 | tr\|G9BRW2\|G9BRW2_DUGJA |
| Pertussis | ko05133 | tr\|E5Q336\|E5Q336_DUGJA |
| Glycolysis / Gluconeogenesis | ko00010 | tr\|D0VYP9\|D0VYP9_DUGJA |
| Cardiac muscle contraction | ko04260 | tr\|O96062\|O96062_DUGJA;  tr\|O96063\|O96063_DUGJA |
| HIF-1 signaling pathway | ko04066 | tr\|D0VYP9\|D0VYP9_DUGJA |
| Shigellosis | ko05131 | tr\|K7X7R0\|K7X7R0_DUGJA |
| NOD-like receptor signaling pathway90 kDa heat shock protein | ko04621 | tr\|C0KTP7\|C0KTP7_DUGJA |
| Measles | ko05162 | tr\|B0LVF7\|B0LVF7_DUGJA |
| Synaptic vesicle cycle | ko04721 | tr\|A5HUF0\|A5HUF0_DUGJA |
| Metabolism of xenobiotics by cytochrome P450 | ko00980 | tr\|G9BRW2\|G9BRW2_DUGJA |
| Progesterone-mediated oocyte maturation | ko04914 | tr\|C0KTP7\|C0KTP7_DUGJA |
| Vasopressin-regulated water reabsorption | ko04962 | tr\|A0A0G4DCR1\|A0A0G4DCR1_DUGJA |
| Long-term depression | ko04730 | tr\|R9S1J3\|R9S1J3_DUGJA |
| Protein digestion and absorption | ko04974 | tr\|O76154\|O76154_DUGJA |
| Ribosome biogenesis in eukaryotes | ko03008 | tr\|D5JG38\|D5JG38_DUGJA |
| Toxoplasmosis | ko05145 | tr\|B0LVF7\|B0LVF7_DUGJA |
| Glutathione metabolism | ko00480 | tr\|G9BRW2\|G9BRW2_DUGJA  tr\|M9NTD0\|M9NTD0_DUGJA |
| Epstein-Barr virus infection | ko05169 | tr\|B0LVF7\|B0LVF7_DUGJA |
| Pyrimidine metabolism | ko00240 | tr\|A0A068AVN2\|A0A068AVN2_DUGJA |
| Glutamatergic synapse | ko04724 | tr\|R9S1J3\|R9S1J3_DUGJA |
| Phospholipase D signaling pathway | ko04072 | sp\|P91924\|ARF_DUGJA |
| Phototransduction | ko04744 | tr\|E5Q336\|E5Q336_DUGJA |
| Proximal tubule bicarbonate reclamation | ko04964 | tr\|O76154\|O76154_DUGJA |
| Lysosome | ko04142 | tr\|A5HUF0\|A5HUF0_DUGJA |
| Glioma | ko05214 | tr\|E5Q336\|E5Q336_DUGJA |
| Olfactory transduction | ko04740 | tr\|E5Q336\|E5Q336_DUGJA |
| Carbon metabolism | ko01200 | tr\|D0VYP9\|D0VYP9_DUGJA |
| Drug metabolism - cytochrome P450 | ko00982 | tr\|G9BRW2\|G9BRW2_DUGJA |
